# Pregnancy Interventions to Improve Birth Outcomes: What Are the Effects on Maternal Outcomes? A Scoping Review

**DOI:** 10.3389/ijph.2022.1604620

**Published:** 2022-11-02

**Authors:** Eleonor Zavala, Mary Rhodes, Parul Christian

**Affiliations:** Department of International Health, Bloomberg School of Public Health, Johns Hopkins University, Baltimore, MD, United States

**Keywords:** pregnancy, maternal health, malnutrition, infectious disease, prenatal care

## Abstract

**Objectives:** Interventions in pregnancy are commonly evaluated for their effects on birth outcomes because maternal infection and poor nutrition are the primary contributors to adverse pregnancy outcomes, especially in low- and middle-income countries (LMICs). However, the extent to which such interventions directly impact maternal health and nutrition has not been succinctly characterized.

**Methods:** We conducted a scoping review of systematic reviews and meta-analyses of 27 pregnancy interventions to summarize the evidence of impact on maternal outcomes.

**Results:** Overall, these were reported incompletely, and we failed to find any evidence for eight interventions. Influenza vaccination, insecticide-treated bed nets, intermittent preventive treatment for malaria, anthelmintic therapy, and treatment of bacterial vaginosis, asymptomatic bacteriuria, and periodontal disease during pregnancy provided direct benefit to women, with reductions in infection risk. Nutritional interventions such as micronutrient supplementation and balanced energy and protein improved outcomes of maternal anemia and gestational weight gain, particularly in deficient populations. Calcium and low dose aspirin significantly reduced the risk of pre-eclampsia.

**Conclusion:** These findings highlight antenatal interventions benefitting maternal health and provide insights into pathways for impacting birth and infant outcomes.

## Introduction

Maternal mortality and morbidity remain a challenge to promoting global health and achieving health equity. In 2017, an estimated 295,000 women died in pregnancy and childbirth, 94% of which were in low- and middle-income countries (LMICs) (1). While global maternal mortality fell by 42.9% between 1990 and 2015, accelerated progress, particularly in low-income countries, is needed to achieve the SDG goal of 70 deaths per 100,000 live births by 2030 (2). The leading causes of maternal death are largely preventable and include obstetric hemorrhage, hypertensive disorders in pregnancy, sepsis or infection, embolism, obstructed labor, and abortion-related complications (3). Contributors to these causes include individual level risk factors, such as infection, poor nutrition, education, and obstetric history, but also broader social, economic, environmental and health systems determinants (4).

Pregnancy increases susceptibility for various infections, including influenza, malaria, and reproductive tract infections (5). In malaria-endemic regions of Africa, one in four pregnant women are estimated to have a malarial infection at the time of delivery, resulting in high risks for adverse birth outcomes and potential for transmission to the child (6). While the prevalence of helminthic infections varies by region, it can be a significant contributor to maternal iron-deficiency anemia (7). Other infections, such as sexually transmitted diseases, bacterial vaginosis, and asymptomatic bacteriuria are also common in pregnancy, with higher rates in LMICs (8, 9). Inflammation and infection in pregnancy can impact fetal development and play a role in the etiology of adverse birth outcomes, including prematurity (10).

Maternal undernutrition represents one of the most prevalent burdens among pregnant women in LMICs (11). Underweight, defined as body mass index (BMI) less than 18.5 kg/m^2^, affected 14.2% of women aged 20–49 years in LMICs in 2015, with prevalence as high as 42% in India in 2014. Anemia in pregnancy, defined as hemoglobin less than 110 g/L, affected 40.1% of pregnancies in 2016. The prevalence of other maternal micronutrient deficiencies is not well documented, but evidence of improved maternal micronutrient status and birth outcomes following supplementation suggests deficiencies are common (11). Maternal undernutrition, including inadequate gestational weight gain, has direct impacts on the growth and development of the fetus, resulting in babies being born too soon and/or too small (12, 13). In LMICs, maternal nutritional and infection-related risk factors often coexist, resulting in synergistic effects, further underscoring the need for integrative antenatal care services (14).

Interventions administered in pregnancy provide the opportunity to lift children out of the intergenerational effects of poor health. As such, many evidence-based interventions and recommendations during pregnancy are targeted at reducing adverse birth outcomes, such as low birth weight (LBW), preterm birth (PTB), small for gestational age (SGA), stillbirth, and neonatal mortality (15). In trials generating the evidence of benefit, outcomes in the newborn are examined primarily, whereas those in the mother are considered secondary and are measured and reported with less frequency (15). Understanding the physiological impact of interventions on the mother can elucidate the mechanisms that promote better fetal development and what factors moderate its efficacy. Furthermore, full knowledge of intervention effects is necessary to ensure they are, at minimum, safe for pregnant women and, ideally, contribute to improvements in maternal health. The objective of this review is to present the evidence on maternal outcomes of those interventions administered in pregnancy where the primary aim is improving birth outcomes.

## Methods

We conducted a literature review of peer-reviewed systematic reviews (SRs), meta-analyses (MAs), and reviews of reviews of select interventions (*n* = 27) during pregnancy that focused on infection prevention and management, and nutritional support in LMIC settings with a focus on maternal health and nutrition outcomes. The selected interventions were informed by a parallel review by Ashorn et al. focusing on birth outcomes (Ashorn, P., personal communication). We did not include antiretroviral therapy for HIV-1 in pregnancy as it has been tested for maternal infection and mother-to-child transmission outcomes.

Outcomes of interest included maternal mortality and morbidity such as hemorrhage, hypertensive disorders, anemia, infections, micronutrient status indicators, adverse events, side effects, and others. Unspecified outcomes were also considered if found in the literature. We did not apply any restrictions to outcome definitions and report the definitions used by the authors when provided.

We conducted individual searches for each intervention in four databases: PubMed, Embase, Cochrane Database of Systematic Reviews, and Scopus. Each search consisted of intervention-related terms, pregnancy terms, and study type specification, i.e., review articles. The search was limited to reviews written in English and Spanish and published from 1950 onwards and last updated on January 25th, 2021. Search terms were restricted to title, abstract, and keywords to obtain the most relevant results. An example of our search strategy is available in Supplementary Table S1.

Two authors (EZ, MR) reviewed the articles independently, using Covidence software. Titles and abstracts were screened, and relevant articles underwent full-text review. We included systematic reviews and meta-analyses that evaluated the intervention of interest and reported at least one maternal outcome. We excluded narrative or scoping reviews, clinical guidelines, and intervention trials in non-pregnant women.

The following information was extracted from the review articles: title, author, year published, review type (SR/MA), effect sizes of pooled data (for MAs) or synthesis of findings (for SRs) by outcome, number of studies and participants, intervention and comparator details, and quality assessment by review authors. We did not conduct an independent quality assessment of the included review articles but report on the published assessments.

## Results

We screened 3,983 articles, conducted 481 full text reviews, and extracted data from 102 systematic reviews or meta-analyses across all interventions (Supplementary Table S2). To avoid duplication, we present the most recent and comprehensive findings from 24 reviews across infection-related interventions (Table 1), nutritional interventions (Table 2) and other interventions (Table 3). Relevant articles were not identified for eight interventions, specifically maternal Hib vaccination, WASH interventions, IPTp plus antibiotics, the screening and treatment of STIs other than HIV and syphilis, the treatment of deep caries of periapical periodontal disease, the screening and treatment of tuberculosis, unconditional cash transfers, and the monitoring of gestational weight gain in undernourished settings. For the other 19 interventions, at least one relevant SR/MA was identified with maternal outcomes. Additional intervention details and the published risk of bias and quality of evidence are provided (Supplementary Table S3).

**TABLE 1:** Infection-related interventions in pregnancy and maternal outcomes (global, 2021).

References	Maternal outcome	Intervention/Comparator	Effect size (95% CI)	Studies, participants (n)
**Influenza virus vaccination administered during pregnancy**				
(16)	Laboratory confirmed influenza (LCI)^a^	Seasonal influenza vaccine vs. saline placebo or meningococcal or pneumococcal vaccine^b^	RR: 0.47 (0.31, 0.71)	3 RCTs, *n* = 10,123
	Influenza-like illness (ILI)^c^		RR: 0.94 (0.85, 1.03)	3 RCTs, *n* = 10,123
	Any respiratory illness (RI)^d^		RR: 0.89 (0.75, 1.05)	2 RCTs, *n* = 2,577
(17)	Maternal death	Seasonal influenza vaccine vs. saline placebo	IRR: 0.80, (0.21, 2.96)	2 RCTs, *n* = 5,809
**Tetanus-Diphtheria-acellular Pertussis (Tdap) vaccination during pregnancy**				
(22)	Hypertension^e^	Tdap vs. no Tdap	RR: 1.02 (0.88, 1.19)	1 RCS, *n* = 68,550
			RR: 1.15 (0.51, 2.61)	1 PCS, *n* = 98
	Pre-eclampsia^f^	Tdap vs. no Tdap/Td	RR: 0.85 (0.69, 1.04)	1 RCS, *n* = 68,550
			RR: 0.51 (0.05, 5.61)	1 RCT, *n* = 272
			RR: 1.40 (0.88, 2.25)	1 PCS, *n* = 98
	Severe pre-eclampsia^g^	Tdap vs. no Tdap	RR: 0.61 (0.39, 0.94)	1 RCS, *n* = 68,550
	Chorioamnionitis^h^	Tdap vs. no Tdap	RR: 1.53 (0.80, 2.90)	6 RCS, *n* = 1,759
			RR: 1.19 (1.13, 1.26)	*n* = 123,494
			RR: 1.14 (1.10, 1.18)	*n* = 994,957
			RR: 1.23 (1.17, 1.28)	*n* = 197,564
			RR: 1.10 (0.70, 1.75)	*n* = 68,550
			RR: 1.51 (0.77, 2.96)	*n* = 7,378
**Provision of insecticide-treated bed nets (ITNs) in pregnancy**				
(24)	Placental malaria^i^	ITNs vs. untreated nets or no nets	RR: 0.77 (0.66, 0.90)	3 RCTs, *n* = 4,457
	Hemoglobin (g/L)		MD: 0.50 g/L (−0.95, 1.95)	4 RCTs, *n* = 6,418
**Changing a two-dose intermittent preventative treatment in pregnancy (IPTp) regimen to more frequent IPTp dosing**				
(25)	Hemoglobin (g/dl)	3 or more doses of IPTp-SP vs. 2 doses		
	*Overall*		MD: 0.13 (0.03, 0.22)	7 RCTs, *n* = 4,216
	*HIV+*		MD: 0.11 (−0.15, 0.37)	4 RCTs, *n* = 676
	*HIV-*		MD: 0.15 (0.04, 0.26)	5 RCTs, *n* = 2,856
	*G1-G2* ^j^		MD: 0.17 (0.04, 0.30)	7 RCTs, *n* = 2,711
	Anemia (<11 g/dl)^k^		RR: 0.95 (0.90, 1.01)	7 RCTs, *n* = 4,216
	Moderate/severe anemia (<8,7, or 6 g/dl)			
	*Overall*		RR: 0.73 (0.48, 1.11)	6 RCTs, *n* = 4,478
	*G1-G2*		RR: 0.60 (0.36, 0.99)	6 RCTs, *n* = 3,130
	Maternal parasitemia^l^			
	*Overall*		RR: 0.68 (0.52, 0.89)	7 RCTs, *n* = 4,218
	*HIV+*		RR: 0.26 (0.15, 0.46)	4 RCTs, *n* = 666
	*HIV-*		RR: 0.86 (0.74, 1.01)	5 RCTs, *n* = 2,852
	*G1-G2*		RR: 0.54 (0.37, 0.80)	7 RCTs, *n* = 2,685
	Placental malaria^m^			
	*Overall*		RR: 0.51 (0.38, 0.68)	6 RCTs, *n* = 2,882
	*HIV+*		RR: 0.38 (0.21, 0.69)	4 RCTs, *n* = 658
	*HIV-*		RR: 0.57 (0.39, 0.82)	4 RCTs, *n* = 1,535
	*G1-G2*		RR: 0.50 (0.35, 0.70)	6 RCTs, *n* = 2,126
**Changing the IPTp regimen from sulfadoxine-pyrimethamine (SP) to dihydroartemisinin-piperaquine (DP)**				
(26)	Clinical malaria during pregnancy^n^	3-dose IPTp-DP vs. standard IPTp-SP	OR: 0.17 (0.10, 0.29)	1 RCT, *n* = 200
		Monthly DP vs. standard IPTp-SP	OR: 0.01 (0.00, 0.19)	1 RCT, *n* = 206
	Placental malaria^o^	3-dose IPTp-DP vs. standard IPTp-SP	OR: 0.73 (0.50, 1.06)	2 RCTs, *n* = 1,231
		Monthly DP vs. standard IPTp-SP	OR: 0.41 (0.23, 0.74)	1 RCT, *n* = 206
	Maternal peripheral malaria^p^	3-dose IPTp-DP vs. standard IPTp-SP	OR: 0.27 (0.15, 0.47)	2 RCTs, *n* = 1,231
		Monthly DP vs. standard IPTp-SP	OR: 0.09 (0.01, 1.68)	1 RCT, *n* = 206
	Anemia (<11 g/dl)	3-dose IPTp-DP versus standard IPTp-SP	OR: 0.75 (0.60, 0.94)	1 RCT, *n* = 1,031
		Monthly DP versus standard IPTp-SP	OR: 0.58 (0.39, 0.84)	1 RCT, *n* = 527
	Maternal SAEs^q^	3-dose IPTp-DP versus standard IPTp-SP	OR: 0.42 (0.29, 0.62)	2 RCTs, *n* = 1,231
		Monthly DP versus standard IPTp-SP	OR: 0.69 (0.19, 2.54)	1 RCT, *n* = 206
**Replacement of IPTp with ISTp (intermittent screening and treatment)**				
(27)	Maternal parasitemia in pregnancy or at delivery^r^	ISTp-ACT vs. IPTp-SP	RR: 1.09 (1.02, 1.17)	4 RCTs, *n* = 7,225
(26)	Clinical malaria during pregnancy	ISTp-DP vs. IPTp-SP	OR: 1.15 (0.87, 1.51)	2 RCTs, *n* = 803
	Placental malaria		OR: 1.29 (1.10, 1.50)	2 RCTs, *n* = 2,903
	Maternal peripheral malaria		OR: 1.39 (1.14, 1.69)	2 RCTs, *n* = 2,903
	Anemia (<11 g/dl)		OR: 0.88 (0.74, 1.04)	2 RCTs, *n* = 2,903
	Maternal SAEs		OR: 0.96 (0.74, 1.24)	2 RCTs, *n* = 2,903
**Preventive anthelmintic treatment in pregnancy**				
(28)	Anemia at term (Hb < 11 g/dl)	Any anthelmintic drug vs. placebo or no treatment	RR: 0.77 (0.73, 0.81)	3 RCTs, *n* = 5,216
	Infection intensity: *T. trichiura* ^s^		RR: 0.69 (0.42, 1.13)	2 RCTs, *n* = 2,867
	Infection intensity: Hookworm^t^		RR: 0.52 (0.18, 1.47)	2 RCTs, *n* = 2,867
**Clindamycin or metronidazole treatment of pregnant women with current bacterial vaginosis (BV)**				
(29)	Failure of test of cure (BV detected)^u^	Any antibiotic^v^ vs. placebo	RR: 0.42 (0.31, 0.56)	10 RCTs, *n* = 4,403
		Clindamycin (vaginal) vs. placebo	RR: 0.40 (0.30, 0.53)	3 RCTs, *n* = 1,411
		Metronidazole (oral) vs. placebo	RR: 0.52 (0.30, 0.88)	3 RCTs, *n* = 2,116
	*Previous preterm birth*	Any antibiotic vs. placebo	RR: 0.57 (0.22, 1.50)	2 RCTs, *n* = 276
	Postpartum infection^w^	Clindamycin or metronidazole vs. placebo	RR: 0.91 (0.26, 3.21)	2 RCTs, *n* = 618
	PPROM	Metronidazole (oral) vs. placebo	RR: 0.74 (0.30, 1.84)	2 RCTs, *n* = 493
	Side effects sufficient to stop treatment	Any antibiotic vs. placebo/no treatment	RR: 1.66 (1.02, 2.68)	4 RCTs, *n* = 2,235
	Side effects not sufficient to stop treatment	Any antibiotic vs. placebo/no treatment	RR: 1.27 (0.76, 2.13)	3 RCTs, *n* = 1,340
**Screening and treatment of asymptomatic bacteriuria (ASB) in pregnancy**				
(32)	Pyelonephritis^x^	Screening vs. no screening	RR: 0.28 (0.15, 0.54)	3 RCS, *n* = 5,659
(31)	Pyelonephritis	Any antibiotic vs. placebo/no treatment	RR: 0.24 (0.13, 0.41)	12 RCTs, *n* = 2,017
	Persistent bacteriuria^y^		RR: 0.30 (0.18, 0.53)	4 RCTs, *n* = 596
**Treatment of documented periodontal disease during pregnancy**				
(33)	Periodontal outcomes^z^	Periodontal treatment vs. no treatment	Not available. Lowest and highest MD included	
	Probing depth		MD: −0.88 (−0.95, −0.81)	3 RCTs, *n* = 1,241
			MD: −0.40 (−0.70, −0.10)	
	Bleeding on probe		MD: −47.6 (−49.6, −45.6)	5 RCTs, *n* = 2,278
			MD: 20.6 (18.7, 22.5)	
	Plaque index		MD: −50.1 (−51.6, −48.5)	2 RCTs, *n* = 1,211
			MD: −43.5 (−47.0, −39.9)	
	Clinical attachment level		MD: −0.80 (−0.90, −0.70)	3 RCTs, *n* = 1,241
			MD: −0.25 (−0.30, −0.20)	
(34)	Pre-eclampsia^aa^	Periodontal treatment vs. to no treatment	RR: 0.98 (0.77, 1.26)	6 RCTs, *n* = 4,397
	Cesarean section		RR: 0.84 (0.67, 1.07)	2 RCTs, *n* = 1,177
	Gestational diabetes		RR: 1.60 (0.64, 4.00)	1 RCT, *n* = 67

^a^ Laboratory confirmed influenza by polymerase chain reaction (PCR).

^b^ In all trials the influenza vaccine was a trivalent inactivated influenza vaccine. In one trial, the control was a quadrivalent meningococcal conjugate vaccine and in another, the control was a 23-valent pneumococcal polysaccharide vaccine.

^c^ Influenza-like illness not defined.

^d^ Respiratory illness with or without fever (>38°C).

^e^ Definition for hypertension varied by study.

^f^ Definition for pre-eclampsia varied by study.

^g^ Severe pre-eclampsia defined by ICD 10-AM O14.1.

^h^ Definition for chorioamnionitis varied by study.

^i^ Placental malaria defined as the presence of asexual parasitemia detectable by microscopy.

^j^ G1-G2 indicates primi- and secundi-gravidae i.e., first or second pregnancies.

^k^ Anemia (<11 g/dl) and severe anemia (defined by individual trials as Hb <6, 7, or 8 g/dl) at term or delivery.

^l^ Maternal malaria infection identified in peripheral blood at delivery.

^m^ Placental malaria (all species) identified by microscopy.

^n^ Clinical malaria episode defined as presence of asexual parasites and fever during pregnancy.

^o^ As defined by individual study authors.

^p^ Parasitemia at delivery, defined by individual study authors.

^q^ Serious adverse events (SAEs) as defined by individual study authors.

^r^ Maternal parasitemia in pregnancy or at term not defined.

^s^ Any T.Trichiura infection, as defined by individual study authors.

^t^ Any hookworm infection, as defined by individual study authors.

^u^ Timing and method of test of cure varied by individual studies. Diagnosis of BV also varied by study (Amsel or clinical criteria, Gram stain criteria, and abnormal Nugent score 4–10).

^v^ Antibiotics included: oral amoxicillin, oral and vaginal clindamycin, oral metronidazole, and oral erythromycin.

^w^ As defined by individual study authors.

^x^ Pyelonephritis (kidney infection) not defined.

^y^ Defined as bacteriuria persisting at the time of delivery.

^z^ Periodontal outcomes included probing depth, plaque index, bleeding on probe, and clinical attachment level as defined by individual study authors.

^aa^ Pre-eclampsia and gestational diabetes were not defined.

RR, relative risk; OR, odds ratio; MD, mean difference; IRR, incidence rate ratio; RCT, randomized-controlled trial; PCS, prospective cohort study; RCS, retrospective cohort study; LCI, laboratory-confirmed influenza; ILI, influenza-like illness; RI, respiratory illness; Tdap, tetanus-diphtheria-acellular pertussis vaccine; Td, tetanus-diphtheria vaccine; TT, tetanus toxoid vaccine; ITN, insecticide-treated bed net; IPTp, intermittent preventative treatment in pregnancy; HIV, human immunodeficiency virus; SP, sulfadoxine-pyrimethamine; DP, dihydroartemisinin-piperaquine; SAE, serious adverse event; ISTp, intermittent screening and treatment in pregnancy; ACT, artemisinin-based combination therapy; BV, bacterial vaginosis; PPROM, preterm pre-labor rupture of membranes; PROM, pre-labor rupture of membranes; PTB, preterm birth; ASB, asymptomatic bacteriuria; LMICs, low-and middle-income countries.

**TABLE 2:** Nutrition interventions in pregnancy and maternal outcomes (global, 2021).

Ref	Maternal outcome	Intervention/Comparator	Effect size (95% CI)	Studies, participants (n)
**Nutrition education for undernourished pregnant women**				
(35)	Protein intake (g/day)	Nutrition education vs. no counseling	MD: 6.99 g/day (3.02, 10.97)	3 RCTs, *n* = 632
	Energy intake (kcal/day)		MD: 105.61 (−18.94, 230.15)	3 RCTs, *n* = 342
	Gestational weight gain (kg)		MD: −0.41 (−4.41, 3.59)	2 RCTs, *n* = 233
(36)	Gestational weight gain (kg)	Any nutrition counseling^a^ vs. standard of care		
		*Overall*	MD: 0.45 (0.12, 0.79)	13 studies^b^, *n* = 2,833
		*LMICs only*	MD: −0.06 (−1.12, 1.01)	3 studies, *n* = 1,307
		Only nutrition counseling vs. standard of care	MD: −0.07 (−1.29, 1.16)	3 studies, *n* = 872
		Nutrition counseling + nutrition support vs. standard of care	MD: 0.15 (0.0, 0.29)	6 studies, *n* = 1,556
	Anemia in 3rd trimester or at delivery^c^	Any nutritional counseling vs. standard of care		
		*Overall*	RR: 0.70 (0.58, 0.84)	11 studies, *n* = 2,588
		*LMICs only*	RR: 0.69 (0.56, 0.85)	8 studies, *n* = 1,942
		Only nutrition counseling vs. standard of care	RR: 0.84 (0.70, 1.00)	4 studies, *n* = 574
		Nutrition counseling + nutrition support vs. standard of care	RR: 0.58 (0.44, 0.76)	4 studies, *n* = 1,359
**Provision of proteins and energy to undernourished pregnant women**				
(35)	Weekly gestational weight gain (g/week)	Balanced protein energy supplementation^d^ vs. control/no intervention	MD: 18.63 g/wk (-1.81, 39.07)	9 RCTs, *n* = 2,391
(37)			MD: 20.74 g/wk (1.46, 40.02)	10 RCTs, *n* = 2,571
(35)	Pre-eclampsia^e^		RR: 1.48 (0.82, 2.66)	2 RCTs, *n* = 463
(37)			RR: 1.20 (0.77, 1.89)	3 RCTs, *n* = 516
**Iron or iron-folic acid (IFA) supplementation**				
(38)	Anemia at term (Hb <110 g/L)	Supplement with iron vs. w/o iron or placebo/no treatment	RR: 0.30 (0.19, 0.46)	14 RCTs, *n* = 2,199
		Supplement with iron and folic acid vs. w/o IFA or placebo/no treatment	RR: 0.34 (0.21, 0.54)	3 RCTs, *n* = 346
	Iron deficiency at term	Supplement with iron vs. w/o iron or placebo/no treatment	RR: 0.43 (0.27, 0.66)	7 RCTs, *n* = 1,256
	Severe anemia (Hb <70 g/L)		RR: 0.22 (0.01, 3.20)	9 RCTs, *n* = 2,125
	Any side effects		RR: 1.29 (0.83, 2.02)	11 RCTs, *n* = 2,423
(39)	Hemoglobin concentration (g/L)	Iron supplement vs. placebo/no iron	MD: 7.80 g/L (4.08, 11.52)	11 studies, *n* = 17,288
		IFA vs. FA	MD: 6.95 g/L (2.80, 11.1)	7 studies, *n* = 16,089
	Serum/plasma ferritin concentration (ug/L)	Iron supplement vs. placebo/no iron	MD: 24.14 ug/L (10.83, 37.45)	9 studies, *n* = 5,045
		IFA vs. FA	MD: 15.87 ug/L (2.96, 28.79)	5 studies, *n* = 3,894
	Pre-eclampsia or eclampsia	Iron supplement vs. placebo/no iron	RR: 1.55 (0.91, 2.63)	3 studies, *n* = 2,773
**Replacement of iron-folic acid (IFA) with multiple-micronutrient (MMS) supplementation**				
(41)	Anemia (Hb <110 g/L) in the 3rd trimester	MMS with IFA vs. Iron, with or w/o folic acid^f^	RR 1.04 (0.94, 1.15)	9 RCTs, *n* = 5,912
	Maternal death		RR: 1.06 (0.72, 1.54)	6 RCTs, *n* = 106,275
(39)	Serum/plasma retinol concentration (umol/L)	MMS with IFA vs. Iron, with or w/o folic acid	MD: 0.11 (0.05, 0.17)	7 studies, *n* = 3,111
	Serum/plasma zinc concentration (nmol/L)		MD: 0.40 (0.18, 0.62)	5 studies, *n* = 3,028
	Serum/plasma vitamin B12 concentration (pmol/L)		MD: 14.77 (5.13, 24.42)	3 studies, *n* = 962
**Provision of lipid-based nutrient supplements (LNS) instead of MMS or IFA**				
(44)	Weekly gestational weight gain (g/week)	LNS vs. IFA	MD: 0.46 SD (−0.44, 1.36)	2 RCTs, *n* = 3,539
		LNS vs. MMS	No difference in GWG between groups	1 RCT, *n* = 662
	Anemia at term or near term (Hb < 110 g/L)	LNS vs. IFA	RR: 2.35, (1.67, 3.30)	1 RCT, *n* = 536
		LNS vs. MMS	RR: 1.40, (1.07, 1.82)	1 RCT, *n* = 557
	Maternal death	LNS vs. IFA	RR: 0.53 (0.12, 2.41)	3 RCTs, *n* = 5,628
**Calcium Supplementation**				
(46)	High blood pressure^g^	High dose calcium^h^ vs. placebo	RR: 0.65 (0.53, 0.81)	12 RCTs, *n* = 15,470
		Low dose calcium^i^ vs. placebo/no treatment	RR: 0.53 (0.38, 0.74)	5 trials, *n* = 665
	Pre-eclampsia^j^	High dose calcium vs. placebo	RR: 0.45 (0.31, 0.65)	13 RCTs, *n* = 15,730
		Low dose calcium vs. placebo/no treatment	RR: 0.38 (0.28, 0.52)	9 trials, *n* = 2,234
		High dose vs. low dose calcium	RR: 0.42 (0.18, 0.96)	1 trial, *n* = 262
	Eclampsia^k^	High dose calcium vs. placebo	RR: 0.73 (0.41, 1.27)	3 RCTs, *n* = 13,425
		Low dose calcium vs. placebo/no treatment	RR: 0.17 (0.01, 4.06)	1 trial, *n* = 168
		High dose vs. low dose calcium	RR: 0.32 (0.07, 1.53)	1 trial, *n* = 262
	Maternal death or serious morbidity^l^	High dose calcium vs. placebo	RR: 0.80 (0.66, 0.98)	4 RCTs, *n* = 9,732
	Placental abruption^m^	High dose calcium vs. placebo	RR: 0.86 (0.55, 1.34)	5 RCTs, *n* = 14,336
		Low dose calcium vs. placebo/no treatment	RR: 1.00 (0.14, 6.90)	3 trials, *n* = 160
	Cesarean section	High dose calcium vs. placebo	RR: 0.95 (0.89, 1.02)	8 RCTs, *n* = 15,234
		Low dose calcium vs. placebo/no treatment	RR: 0.73 (0.46, 1.15)	4 trials, *n* = 521
	HELLP syndrome^n^	High dose calcium vs. placebo	RR: 2.67 (1.05, 6.82)	2 RCTs, *n* = 12,901
**Supplementation with omega-3 fatty acids**				
(48)	High blood pressure^o^	Omega-3 LCPUFA^p^ (supplements or food) vs. placebo or no omega-3	RR: 1.03 (0.89, 1.20)	7 RCTs^q^, *n* = 4,531
	Pre-eclampsia^r^		RR: 0.84 (0.69, 1.01)	20 RCTs, *n* = 8,306
	Eclampsia^s^		RR: 0.14 (0.01, 2.70)	1 RCT, *n* = 100
	Gestational diabetes^t^		RR: 1.02 (0.83, 1.26)	12 RCTs, *n* = 5,235
	Anemia^u^		RR: 1.16 (0.91, 1.48)	1 RCT, *n* = 846
	Gestational weight gain (kg)		MD: −0.05 kg (−0.68, 0.59)	11 RCTs, *n* = 2,297
	PPROM^v^		RR: 0.53 (0.25, 1.10)	3 RCTs, *n* = 925
	PROM		RR: 0.41 (0.21, 0.82)	3 RCTs, *n* = 915
	Any adverse event^w^		RR: 1.38 (1.16, 1.65)	5 RCTs, *n* = 1,480
	Serious adverse event^x^		RR: 1.04 (0.40, 2.72)	2 RCTs, *n* = 2,690
	Maternal death		RR: 1.69 (0.07, 39.30)	4 RCTs, *n* = 4,830
	Cesarean section		RR: 0.97 (0.91, 1.03)	28 RCTs, *n* = 8,481
	Postpartum hemorrhage^y^		RR: 1.03 (0.82, 1.30)	4 RCTs, *n* = 4,085
	Postpartum depression^z^		RR: 0.99 (0.56, 1.77)	2 RCTs, *n* = 2,431

^a^ Any nutrition counseling interventions including those with additional health messages and/or with nutrition support, such as food or micronutrient supplements.

^b^ Study designs included RCTs, cluster-RCTs, and quasi-experimental (nonrandomized) designs. Characterization of study design in meta-analyses was not reported.

^c^ Anemia not defined.

^d^ Balanced protein-energy supplementation was defined as nutritional supplementation during pregnancy in which protein provided less than 25% of total energy content.

^e^ In Ota et al., pre-eclampsia was defined by individual study authors. In Imdad & Bhutta, pre-eclampsia was not defined.

^f^ Two trials in the review used iron without folic acid as the controls, whereas the remaining trials (17) used IFA.

^g^ High blood pressure as defined by individual study authors, with or without proteinuria.

^h^ High dose calcium defined as ≥1 g of dietary calcium.

^i^ Low dose calcium defined as <1 g of dietary calcium.

^j^ High blood pressure with significant proteinuria, as defined by individual study authors.

^k^ Eclampsia not defined.

^l^ Composite outcome of death or at least one measure of serious morbidity: eclampsia; renal failure; HELLP syndrome; and admission to intensive care.

^m^ Placental abruption not defined.

^n^ HELLP syndrome defined as syndrome of hemolysis, elevated liver enzymes, and low platelets.

^o^ High blood pressure defined as high blood pressure without proteinuria (no cut-off indicated).

^p^ Omega-3 long-chain polyunsaturated fatty acids (LCPUFA) as supplements or dietary additions.

^q^ Randomized-controlled trials, including quasi-randomized trials.

^r^ Pre-eclampsia defined as hypertension with proteinuria.

^s^ Eclampsia not defined.

^t^ Gestational diabetes not defined.

^u^ Anemia not defined.

^v^ PPROM and PROM not defined.

^w^ Adverse events as defined by individual study authors.

^x^ Serious adverse events not defined.

^y^ Postpartum hemorrhage not defined.

^z^ Assessed using the Edinburgh Postnatal Development Scale (EPDS). Thresholds described as varying per study and defined by individual study authors.

RR, relative risk; MD, mean difference; RCT, randomized-controlled trial; LMICs, low-and middle-income countries; IFA, iron-folic acid; MMN, multiple-micronutrient; BEP, balanced energy-protein supplementation; LNS, lipid-based nutrient supplements; SD, standard deviations; GWG, gestational weight gain; PPROM, preterm pre-labor rupture of membranes; PROM, pre-labor rupture of membranes; EPDS, Edinburgh Postnatal Depression Scale; CCT, conditional cash transfer.

**TABLE 3:** Other interventions in pregnancy and maternal outcomes (global, 2021).

References	Maternal outcome	Intervention/Comparator	Effect size/Synthesis (95%CI)	Studies, participants (n)
Provision of aspirin during pregnancy				
(54)	Pre-eclampsia^a^	Antiplatelet agents^b^ vs. placebo/no antiplatelet	RR: 0.82 (0.77, 0.88)	60 RCTs, *n* = 36,716
	High-risk women^c^		RR: 0.90 (0.82, 0.98)	26 RCTs, *n* = 11,076
	Randomization before 20 weeks gestation		RR: 0.86 (0.78, 0.95)	27 RCTs, *n* = 18,950
	Dosage ≥ 75 mg		RR: 0.78 (0.66, 0.92)	16 RCTs, *n* = 9,107
	Eclampsia^d^		RR: 1.09 (0.69, 1.71)	14 RCTs, *n* = 24,742
	Gestational hypertension^e^		RR: 0.95 (0.90, 1.01)	25 RCTs, *n* = 27,834
	Postpartum hemorrhage^f^		RR: 1.06 (1.00, 1.13)	16 RCTs, *n* = 23,396
	Placental abruption		RR: 1.22 (0.95, 1.56)	24 RCTs, *n* = 30,257
	Maternal death		RR: 1.75 (0.51, 5.96)	18 RCTs, *n* = 28,675
	Severe maternal morbidity^g^		RR: 1.00 (0.72, 1.39)	15 RCTs, *n* = 28,065

^a^ Pre-eclampsia as defined by individual study authors.

^b^ Any antiplatelet agent such as low-dose (not defined) aspirin or dipyridamole. Dosage, duration, and mode of administration varied by individual trial.

^c^ Maternal risk of pre-eclampsia as defined by individual study authors.

^d^ Eclampsia not defined.

^e^ Defined as new hypertension with onset after 20 weeks’ gestation, using best available definition for every individual study.

^f^ Postpartum hemorrhage defined as blood loss greater than 500 ml.

^g^ Severe maternal morbidity included eclampsia, liver failure, renal failure, disseminated intravascular coagulation, HELLP syndrome, stroke.

RR, relative risk; RCT, randomized-controlled trial.

### Seasonal Influenza Virus Vaccination

Pregnant women vaccinated with seasonal influenza virus experienced a 53% lower risk of laboratory confirmed influenza compared to those who received a saline placebo (RR: 0.47, 95% CI: 0.3–0.71, 3 RCTs, *n* = 10,123) (Table 1), although reductions in influenza-like illness and any respiratory illness were not statistically significant in the pooled analyses and there were no serious adverse events (16). A pooled analysis of two RCTs found a non-significant reduction in maternal death following influenza vaccination compared to placebo (IRR: 0.80, 95% CI: 0.21, 2.96, 2 RCTs, *n* = 5,809) (17).

### Tetanus-Diphtheria-Pertussis (Tdap) Vaccination

Several systematic reviews have reported on the effectiveness of Tdap vaccination during pregnancy for neonatal outcomes, but only safety indicators were reported for mothers, with no morbidity effects examined (39–42). There was no increased risk for gestational hypertensive disorders post vaccination but higher rates of chorioamnionitis were found in three of six retrospective cohort studies, with significant risk estimates ranging from 1.11 (95% CI: 1.03–1.21) to 1.23 (95% CI: 1.17–1.28), although this was not associated with clinically relevant sequelae, such as preterm birth or ICU admission (Table 1) (18). The overall risk of bias was judged to be serious to critical, in large part due to the limitations of retrospective cohort study designs.

### Insecticide-Treated Bed Nets

While it has been established that ITNs are efficacious at reducing childhood morbidity and mortality, their documented effects in pregnant women have been inconsistent (43). In regions of Africa with stable malaria transmission, ITNs reduced placental parasitemia by 23% in all gravidae (RR: 0.77, 95% CI: 0.66–0.90, 3 RCTs, *n* = 4,457) as compared to no nets (Table 1) (19). However, the effect on maternal hemoglobin was not significant (MD: 0.50 g/L, 95% CI: 0.95-1.95, 4 RCTs, *n* = 6,418) (19).

### Three or More Doses of Intermittent Preventive Treatment in Pregnancy with Sulfadoxine-Pyrimethamine

A SRMA including seven RCTs conducted in malaria-endemic countries of Sub-Saharan Africa found three or more doses of IPTp-SP was associated with a 32% and 49% reduced risk of maternal parasitemia and placental malaria, respectively, compared to two doses (95% CI: 0.52–0.89; 7 RCTs, *n* = 4,218); (95% CI: 0.38, 0.68; 6 RCTs, *n* = 2,882), with greater effects among HIV+ and primi- and secundi-gravidae women (Table 1) (20). More frequent dosing improved hemoglobin by 0.13 g/dl overall and resulted in a 40% reduction of moderate or severe anemia among primi- and secundi-gravidae mothers (RR: 0.60, 95% CI: 0.36–0.99, 6 RCTs, *n* = 3,130) (20). Included trials were classified as high GRADE quality except two due to high risk of bias.

### Dihydroartemisinin-Piperaquine IPTp

Three trials in SSA assessed three-dose or monthly IPTp-DP versus IPTp-SP and found lower odds of clinical malaria (OR: 0.17, 95% CI: 0.10–0.29, 2 RCTs, *n* = 498) as well as placental malaria and peripheral malaria at delivery (Table 1) (21). In individual trials, three-dose IPTp-DP and monthly IPTp-DP reduced the odds of maternal anemia (<11 g/dl) by 25% (95% CI: 0.60–0.94, *n* = 1,031) and 42% (95% CI: 0.39–0.84, *n* = 527) compared to IPTp-SP group, respectively. IPTp-DP was also associated with fewer maternal adverse events compared to IPTp-SP, but with low certainty evidence (21).

### Intermittent Screening and Treatment in Pregnancy

ISTp-ACT (artemisinin-based combination therapy) versus IPTp-SP resulted in a 9% increased risk of maternal parasitemia in the screen and treat groups (RR:1.09, 95% CI: 1.02–1.17, 4 RCTs, *n* = 7,225) (22). In two trials conducted in highly SP-resistant areas in Kenya and Malawi, the pooled analysis showed an increased risk of placental malaria with high certainty evidence (OR: 1.29, 95% CI: 1.10–1.50, *n* = 2,903) (21). Rates of maternal anemia and serious adverse events were not different between groups (Table 1).

### Preventive Anthelmintic Treatment

A pooled analysis found that any anthelmintic drug during pregnancy reduced maternal anemia by 23% (RR: 0.77, 95% CI: 0.73–0.81, 3 RCTs, *n* = 5,216) and judged the evidence as moderate quality (23). However, reductions in hookworm or *T. trichiura* infection density with anthelmintic treatment were not significant (Table 1).

### Antibiotic Treatment for Bacterial Vaginosis

A 2013 Cochrane review summarized the evidence from 21 good quality trials assessing antibiotic treatment, usually clindamycin or metronidazole, compared to placebo or no treatment (24). Overall, antibiotic treatment was found to be effective in clearing BV infection (RR: 0.42, 95% CI: 0.31–0.56, 10 RCTs, *n* = 4,403), but did not reduce risks for postpartum infection or preterm prelabor rupture of membranes (PPROM) (Table 1). Among women who had a previous PTB, antibiotic treatment led to a nonsignificant reduction in BV clearance (RR: 0.57, 95% CI: 0.22–1.50, 2 RCTs, *n* = 276) and did not affect the risk of subsequent PTB (RR: 0.78, 95% CI: 0.42–1.48, 3 RCTs, *n* = 421). Antibiotic treatment also resulted in greater side effects, in some cases sufficient to stop treatment, but the kinds of side effects were not detailed (24). A subsequent review highlighted side effects such as candidal vaginitis, troublesome discharge, and withdrawal due to itching as being more common in antibiotic treatment groups versus placebo groups, however effects were not pooled or consistent across studies (44).

### Screening and Treatment of Asymptomatic Bacteriuria

Treatment of ASB with antibiotics compared to no treatment or placebo was found to reduce the risk of pyelonephritis by 76% (RR: 0.24, 95% CI: 0.13–0.41, 12 RCTs, *n* = 2017), though the evidence was considered low GRADE quality (26). In a SR assessing the effects of ASB screening effectiveness, the risk of pyelonephritis fell by a similar 72% margin in screened versus unscreened pregnancies (RR: 0.28, 95% CI: 0.15–0.54, 3 studies, *n* = 5,659) (25).

### Treatment of Periodontal Disease

A 2017 Cochrane review of periodontal treatment trials in pregnancy found improved periodontal outcomes, including probing depth, bleeding on probe, plaque index, and clinical attachment level (Table 1) (27). A recent SRMA described that periodontal treatment had no effect on the risk of pre-eclampsia (RR: 0.98, 95% CI: 0.77–1.26, 6 RCTs, *n* = 4,397), C-section (RR:0.84, 95% CI: 0.67–1.07, 2 RCTs, *n* = 1,177), or gestational diabetes (RR: 1.60, 95% CI: 0.64–4.00, *n* = 67) (28).

### Nutrition Education in Undernourished Populations

Nutritional counseling increased protein intake in pregnancy (MD: +6.99 g/day, 95% CI: 3.02–10.97), may have increased energy intake (MD: 105.61 kcal/day, 95% CI: −18.94–230.15), and had no effect on gestational weight gain (GWG) (MD: −0.41, 95% CI: −4.41–3.59) as compared to no counseling, but the quality of evidence was deemed as very-low (Table 2) (29). An earlier review found GWG was significantly higher in women who received nutritional counseling than in control groups (MD: 0.45 kg, 95% CI: 0.12–0.79), however, this result was only significant when counseling was accompanied by nutritional support such as food or micronutrient supplements and in studies conducted in high-income settings (Table 2) (30). Nutritional counseling reduced the risk of maternal anemia in the third trimester by 30% (RR: 0.70, 95% CI: 0.58–0.84), including in LMIC settings (RR 0.69, 95% CI 0.56–0.85). When coupled with food or micronutrient supplementation, the effect of nutrition counseling on anemia was more pronounced (RR: 0.58, 95% CI: 0.44–0.76) (30). The quality of evidence for maternal outcomes of weight gain and anemia in this review were considered low.

### Balanced Energy Protein Supplementation

BEP supplementation trials have previously found no increase in weekly GWG (MD: 18.63 g/wk, 95% CI: −1.81, 39.07, 9 RCTs, *n* = 2,391) (29), but a meta-analysis that included one additional trial found a small but significant increase (MD: 20.74 g/wk, 95% CI: 1.46–40.02, 10 RCTs, *n* = 2,571) (31). Neither review found BEP to have any effect on the risk of pre-eclampsia (Table 2).

### Iron and Folic Acid Supplementation

An updated Cochrane review reported daily iron supplementation compared to no iron or placebo decreased maternal anemia by 70% (95% CI: 0.19–0.46, 14 RCTs, *n* = 2,199) and maternal iron deficiency by 57% (95% CI: 0.27–0.66, 7 RCTs, *n* = 1,256) (32). Oh et al. further supported these findings by highlighting the effects of iron supplementation on increasing maternal hemoglobin concentration (MD: 7.80 g/L, 95% CI: 4.08–11.52, 11 studies, *n* = 17,288) and serum/plasma ferritin concentrations (MD: 24.14 μg/L, 95% CI: 10.83–37.45, 9 studies, *n* = 5,045) (33). Improvements in anemia and gains in hemoglobin and ferritin concentrations were similar when IFA supplements were compared to folic acid only or placebo/no treatment (Table 2). Severe anemia (Hb <70 g/L) and pre-eclampsia or eclampsia were not significantly different between iron-containing supplement groups and comparison groups. A separate Cochrane review found that when compared to daily IFA, intermittent IFA did not result in a difference in risk of anemia (RR: 1.22, 95%CI: 0.84–1.80, 4 studies, *n* = 676), though it did result in a decreased risk of side effects (RR: 0.56, 95% CI: 0.37–0.84, 11 studies, *n* = 1777) although the evidence was deemed to be of low to very low quality and several studies were considered to have a high risk of bias (45).

### Multiple Micronutrient Supplementation

Over the past 2 decades, 19 trials comparing MMS versus the standard of care of IFA have been undertaken in LMICs to test the efficacy of the intervention for reducing low birth weight and other adverse outcomes. MMS did not differ compared to IFA with regard to anemia reduction in the third trimester (RR: 1.04, 95% CI: 0.94–1.15, 9 RCTs, *n* = 5,912) nor did it impact maternal mortality (RR: 1.06, 95% CI: 0.72–1.54, 6 RCTs, *n* = 106,275), with evidence of moderate certainty (34). Another SRMA similarly found no differences in maternal mortality or anemia, which could be explained by the lack of differences in plasma hemoglobin, ferritin, transferrin receptor, and folate concentrations between groups (33). However, the pooled analyses did reveal higher levels of serum or plasma retinol, zinc, and vitamin B-12 concentrations in women supplemented with MMS compared to IFA (Table 2) (33). Additional findings from Nepal and Bangladesh found decreases in the prevalence of serum riboflavin, vitamin B-6, vitamin B-12, folate, and vitamin D deficiencies, and in the prevalence of vitamins B-12, A, and D and zinc deficiencies with MMS compared to placebo or IFA, respectively (46, 47). These findings suggest MMS is superior to IFA in improving overall maternal micronutrient status in addition to decreasing adverse birth outcomes.

### Lipid-Based Nutrient Supplements

LNS supplements have been studied as a potential vehicle for MMS in pregnancy. In a recent review of four trials, LNS was not found to increase weekly GWG or reduce maternal mortality when compared to IFA or MMS alone (Table 2) (35).

### Calcium Supplementation

Calcium supplementation during pregnancy reduces the risk of pre-eclampsia by 55% (RR: 0.45, 95% CI: 0.31–0.65, 13 RCTs, *n* = 15,730), with the greatest reductions among populations with low baseline calcium intakes (48). A 2018 update of the Cochrane review found a 35% reduction in high blood pressure, a 55% reduction in pre-eclampsia, and a 20% reduction in the composite outcome of maternal death or serious morbidity when comparing ≥1 g/day of calcium versus placebo (Table 2) (36). In a complementary review, non-hypertension related adverse outcomes, such as C-section, urinary stones, urinary tract infection, anemia, and side effects did not differ between supplemented and placebo or no treatment groups (49). Low dose calcium (less than 1 g per day) compared to placebo or no treatment significantly reduced the risk of high-blood pressure (RR: 0.53, 95% CI: 0.38–0.74, 5 studies, *n* = 665) and pre-eclampsia (RR: 0.38, 95% CI: 0.28–0.52, 9 studies, *n* = 2,234) (36), although the quality of evidence is low. Despite the beneficial effects of low and high dose calcium on hypertension and preeclampsia, the risk of eclampsia was not significantly reduced with either intervention (Table 2).

### Omega-3 Fatty-Acid Supplementation

Evidence from 70 trials of supplementation with omega-3 fatty acids vs. placebo or no treatment found no effect on outcomes, including hypertension, gestational diabetes, weight gain, C-section, and other adverse events (Table 2) (37). The exceptions were prelabor rupture of membranes (PROM) (RR: 0.41, 95% CI: 0.21–0.82, 3 trials, *n* = 915) (37) and preeclampsia (RR: 0.82, 95% CI: 0.70–0.97, 14 RCTs, *n* = 10,806) (50) which were reduced with n-3 fatty acid supplementation.

### Conditional Cash Transfers

CCT programs (*n* = 7) were effective in increasing antenatal care uptake and use of a skilled birth attendant at delivery, which can be arguably considered to provide a health benefit, however no direct effects on maternal health outcomes were identified (51). An earlier review evaluating the *Oportunidades* CCT program in Mexico, with the condition of attending antenatal care found that maternal mortality in areas exposed to the program was 11% lower than in areas that were not exposed from 1995 to 2002 (RR: 0.89, 95% CI: 0.82–0.95) (52, 53). A trial in Nepal examining the effects of participatory learning and action (PLA) women’s groups, alone, with food transfers (fortified flour), or with unconditional cash transfers found that pregnant women in the PLA + cash arm experienced a 0.35 increase in diet diversity score (95%CI: 0.08–0.63, *n* = 789) and an increase in mid-upper arm circumference (MD: 0.75 cm, 95% CI: 0.33–1.17) compared to the control group (54).

### Provision of Aspirin

A 2019 Cochrane review reported with high certainty evidence that any antiplatelet agent versus placebo or no treatment resulted in a 18% reduced risk of pre-eclampsia (RR: 0.82, 95% CI: 0.77–0.88, 60 RCTs, *n* = 36,716) (38). The greatest reductions were observed among women at high risk of pre-eclampsia as defined by individual study authors and when treatment was started before 20 weeks gestation (Table 3). High dose aspirin (≥75 mg) led to great reductions in the risk of pre-eclampsia (RR: 0.78, 95% CI: 0.66–0.92, 16 RCTs, n = 9,107) compared with low-dose (<75 mg) aspirin (RR: 0.92, 95%CI: 0.85–1.00, 11 RCTs, *n* = 22,618). However, the risk of eclampsia was not reduced with aspirin, and the risk of postpartum hemorrhage increased slightly (RR: 1.06, 95% CI:1.00–1.13, 16 RCTs, *n* = 23,396). Other maternal outcomes, such as placental abruption, death, and severe morbidity were not affected by antiplatelet therapy (38). A recent multi-site trial (*n* = 11,976) found administration of low dose (81 mg) aspirin in pregnancy was associated with an 11% reduced risk of preterm birth (RR: 0.89, 95% CI: 0.81–0.98) (55), whereas effects on maternal outcomes of hypertensive disorders, hemorrhage, anemia, and maternal mortality were not significantly different with low dose aspirin. However, hypertensive disorders among women with an early preterm delivery (<34 weeks) had a 62% reduced risk of hypertensive disorders with low dose aspirin (95% CI: 0.17–0.85) (55).

## Discussion

The goal of this scoping review was to present the evidence on the maternal effects of infection-related, nutrition and other interventions administered in pregnancy with the aim of improving birth outcomes. Findings of maternal effects were far less reported nor primary compared to birth and infant outcomes, as demonstrated by the eight interventions where no reviews reporting maternal outcomes could be identified. Despite this lack of data, several important findings can be drawn from this work.

Vaccination of pregnant women against infectious diseases has been predominately evaluated for its effect on young infants, with limited evidence on maternal morbidity effects. However, vaccine use in pregnancy has been shown to protect mothers from infection, including influenza, and more recently, SARS-CoV-2 (56). Tdap vaccination in pregnancy is considered safe for the mother and fetus and should be scaled up globally with continued surveillance of chorioamnionitis outcomes. Additional research is needed to discover more effective vaccines, to characterize the optimal timing and seasonality of vaccination in pregnancy for maternal immunogenicity and protection of the infant, and to identify strategies for increasing coverage and uptake globally (57–60).

Insecticide-treated bed nets and frequent IPTp-SP are effective interventions in reducing maternal malaria infection and transmission. In areas of high SP resistance, transitioning to DP should be considered given its effectiveness in reducing maternal malaria and anemia. Studies of cost-effectiveness will help determine whether this new drug regimen should be widely adopted. Conversely, the intermittent screen and treat approach is not yet a suitable alternative to IPTp, even in SP-resistant areas, because the lack of sensitivity of the rapid diagnostic tests (RDTs) may cause more maternal malaria infections to go untreated.

Anthelmintic treatment in pregnancy is recommended and effective in reducing anemia, but evaluations of large-scale implementation programs could shed more light on the effectiveness of anthelmintic treatment for specific parasites. Additional research on whether two doses result in better outcomes, particularly in highly endemic areas, is needed (61).

Treatment with antibiotics is effective in clearing BV in pregnancy but does not appear to reduce the risk of maternal or infant adverse events. With high rates of BV being reported in LMICs (62), more research is needed to determine whether broad screen and treat programs are effective in these settings, whether specific sub-groups benefit more from treatment, and whether earlier administration of treatment improves outcomes (63). Given the significant increased risk of side effects with antibiotic treatment, exploration of alternatives to antibiotic therapy is warranted (64).

Periodontal treatment in pregnancy ameliorates maternal health through improving dental health, but additional research is needed to understand how periodontal indices may link to fetal growth and birth outcomes, and whether timing and type of treatment plays a role. Treatment of ASB in pregnancy significantly reduces the risk of pyelonephritis, but research on effectiveness and cost-effectiveness of RDTs on maternal outcomes is lacking in LMICs. Similarly, evidence on the effectiveness of screen and treat programs for STIs and TB in pregnancy are needed, particularly in LMICs where burdens are high.

Nutrition interventions more commonly reported direct maternal effects compared to the infection-related interventions. Nutrition education may help undernourished pregnant women improve their dietary intakes, but nutritional support, i.e., provision of supplements or food, may be key to improving nutritional status, particularly in food insecure contexts. While the current WHO recommendation does not promote BEP as an individual level intervention, many women in LMICs may experience low BMI when entering pregnancy, even if 20% of the overall population is not underweight as illustrated by sub-national map of the world for low BMI among women (11), and many more may experience inadequate GWG regardless of their BMI at the start of pregnancy (65). As such, regular screening of GWG has the potential to be an effective intervention to ensure adequate weight gain and dietary intakes, especially if followed by nutritional supplementation such as BEP (12). Exploring this targeted approach to nutrition in antenatal care is pressing as populations across LMICs grow more heterogenous in nutritional status. Trials underway using expert consensus formulations of BEP will provide future evidence for the impact of fortified BEP supplementation on birth and maternal outcomes (NCT03533712, NCT03668977, NCT04012177) (66).

IFA is highly effective in reducing maternal anemia and improving iron status. The replacement of IFA with MMS should be considered based on the evidence regarding improved birth outcomes and maternal micronutrient status with MMS supplementation. Findings from individual trials suggest additional maternal health benefits with MMS supplementation, such as reduced cortisol and erythropoietin levels in the third trimester (67), and reductions in obstetric complications such as PPROM, postpartum hemorrhage, and puerperal sepsis (68).

High dose calcium is recommended for reducing the risk of hypertension and preeclampsia in pregnancy in low calcium intake settings; the cost and adherence issues related to high dosage have limited the scale-up in such settings (69). Ongoing non-inferiority trials of low vs. high dose calcium will shed light on this issue (NCT03725891, NCT03735433).

Supplementation with omega-3 fatty acids has not been strongly associated with any maternal outcomes, though additional research on hypertension-related and metabolic outcomes are indicated (70, 71). RCT evidence is needed to evaluate conditional and unconditional cash transfer programs in pregnancy on maternal outcomes, short and long-term. Implementation research is needed to evaluate how either high or low dose aspirin interventions may be integrated within antenatal care and brought to scale.

Much of the current focus in maternal and child health in LMICs is on halting the intergenerational cycles of poor health, and pregnancy and pre-pregnancy interventions are evaluated by their effects on birth outcomes, particularly gestational age and growth indicators. The likelihood that these indicators will be influenced by a particular intervention relies in large part on the maternal physiological response. Maternal health and nutritional status are often considered ‘secondary’ and are either omitted from research results or never measured in the first place, except in the context of interventions that are directly targeted to impact maternal infection, health and survival. Our review demonstrates the importance of capturing maternal outcomes to elucidate whether and by what extent direct effects on the mother impact her and may in turn be the pathway for influencing fetal and infant outcomes. We found an overall under-reporting of maternal outcomes for the selected interventions, and very few results on maternal mortality and serious morbidity. Some studies reported long-term impacts of pregnancy interventions on children, but no long-term health effects on mothers were described, despite the plausible hypothesis that such effects could accrue. There is also a gap in our understanding of the cost-benefit ratios of interventions, which exclude assessments of benefits to maternal health. Future RCTs should include and measure maternal outcomes as primary and should systematically report non-significant estimates and rare events. In summary, our scoping review of antenatal interventions may help policy makers, program implementers, and researchers identify what interventions should be prioritized for the benefit of maternal health in high burden settings.

## References

[1] World Health Organization. Trends in Maternal Mortality 2000 to 2017 Estimates by WHO, UNICEF, UNFPA, World Bank Group and the United Nations Population Division. Geneva: World Health Organization (2019).

[2] Alkema L Chou D Hogan D Zhang S Moller A-B Gemmill A Global, Regional, and National Levels and Trends in Maternal Mortality between 1990 and 2015, with Scenario-Based Projections to 2030: a Systematic Analysis by the UN Maternal Mortality Estimation Inter-Agency Group. Lancet (2016) 387(10017):462–74. 10.1016/S0140-6736(15)00838-7 26584737 PMC5515236

[3] Say L Chou D Gemmill A Tunçalp Ö Moller A-B Daniels J Global Causes of Maternal Death: a WHO Systematic Analysis. Lancet Glob Health (2014) 2(6):e323–33. 10.1016/S2214-109X(14)70227-X 25103301

[4] Filippi V Chou D Ronsmans C Graham W Say L. Levels and Causes of Maternal Mortality and Morbidity. In: Black RE Laxminarayan R Temmerman M Walker N, editors. Reproductive, Maternal, Newborn, and Child Health: Disease Control Priorities. 3rd ed, Vol. 2. Washington (DC): The International Bank for Reconstruction and Development/The World Bank (2016).

[5] Sappenfield E Jamieson DJ Kourtis AP. Pregnancy and Susceptibility to Infectious Diseases. Infect Dis Obstet Gynecol (2013) 2013:752852. 10.1155/2013/752852 23935259 PMC3723080

[6] Desai M ter Kuile FO Nosten F McGready R Asamoa K Brabin B Epidemiology and burden of Malaria in Pregnancy. Lancet Infect Dis (2007) 7(2):93–104. 10.1016/S1473-3099(07)70021-X 17251080

[7] Ness TE Agrawal V Bedard K Ouellette L Erickson TA Hotez P Maternal Hookworm Infection and its Effects on Maternal Health: A Systematic Review and Meta-Analysis. Am J Trop Med Hyg (2020) 103(5):1958–68. 10.4269/ajtmh.20-0503 32840198 PMC7646767

[8] Chico RM Mayaud P Ariti C Mabey D Ronsmans C Chandramohan D. Prevalence of Malaria and Sexually Transmitted and Reproductive Tract Infections in Pregnancy in Sub-saharan Africa: a Systematic Review. JAMA (2012) 307(19):2079–86. 10.1001/jama.2012.3428 22665107

[9] Gilbert NM O’Brien VP Hultgren S Macones G Lewis WG Lewis AL. Urinary Tract Infection as a Preventable Cause of Pregnancy Complications: Opportunities, Challenges, and a Global Call to Action. Glob Adv Health Med (2013) 2(5):59–69. 10.7453/gahmj.2013.061 24416696 PMC3833562

[10] Cappelletti M Bella SD Ferrazzi E Mavilio D Divanovic S. Inflammation and Preterm Birth. J Leukoc Biol (2016) 99(1):67–78. 10.1189/jlb.3MR0615-272RR 26538528

[11] Victora CG Christian P Vidaletti LP Gatica-Domínguez G Menon P Black RE. Revisiting Maternal and Child Undernutrition in Low-Income and Middle-Income Countries: Variable Progress towards an Unfinished Agenda. The Lancet (2021) 397(10282):1388–99. 10.1016/S0140-6736(21)00394-9 PMC7613170 33691094

[12] Christian P Smith ER Zaidi A. Addressing Inequities in the Global burden of Maternal Undernutrition: the Role of Targeting. BMJ Glob Health (2020) 5(3):e002186. 10.1136/bmjgh-2019-002186 PMC7101036 32231793

[13] Goldstein RF Abell SK Ranasinha S Misso M Boyle JA Black MH Association of Gestational Weight Gain with Maternal and Infant Outcomes: A Systematic Review and Meta-Analysis. JAMA (2017) 317(21):2207–25. 10.1001/jama.2017.3635 28586887 PMC5815056

[14] Claycombe KJ Brissette CA Ghribi O. Epigenetics of Inflammation, Maternal Infection, and Nutrition. J Nutr (2015) 145(5):1109S–1115S. 10.3945/jn.114.194639 25833887 PMC4410493

[15] World Health Organization. WHO Recommendations on Antenatal Care for a Positive Pregnancy Experience. Geneva, Switzerland: World Health Organization (2016). 28079998

[16] Quach THT Mallis NA Cordero JF. Influenza Vaccine Efficacy and Effectiveness in Pregnant Women: Systematic Review and Meta-Analysis. Matern Child Health J (2020) 24(2):229–40. 10.1007/s10995-019-02844-y 31865602

[17] Clark DR Omer SB Tapia MD Nunes MC Cutland CL Tielsch JM Influenza or Meningococcal Immunization during Pregnancy and Mortality in Women and Infants: A Pooled Analysis of Randomized Controlled Trials. Pediatr Infect Dis J (2020) 39(7):641–4. 10.1097/INF.0000000000002629 32379201 PMC7279057

[18] Vygen-Bonnet S Hellenbrand W Garbe E von Kries R Bogdan C Heininger U Safety and Effectiveness of Acellular Pertussis Vaccination during Pregnancy: a Systematic Review. BMC Infect Dis (2020) 20(1):136. 10.1186/s12879-020-4824-3 32054444 PMC7020352

[19] Gamble C Ekwaru PJ Garner P Kuile F. Insecticide-Treated Nets for the Prevention of Malaria in Pregnancy: A Systematic Review of Randomised Controlled Trials. PLOS Med (2007) 4(3):e107. 10.1371/journal.pmed.0040107 17388668 PMC1831739

[20] Kayentao K Garner P Maria van Eijk A Naidoo I Roper C Mulokozi A Intermittent Preventive Therapy for Malaria during Pregnancy Using 2 vs 3 or More Doses of Sulfadoxine-Pyrimethamine and Risk of Low Birth Weight in Africa: Systematic Review and Meta-Analysis. JAMA (2013) 309(6):594–604. 10.1001/jama.2012.216231 23403684 PMC4669677

[21] Olaleye A Okusanya BO Oduwole O Esu E Meremikwu M. A Systematic Review and Meta-Analysis of Dihydroartemisinin-Piperaquine versus Sulphadoxine-Pyrimethamine for Malaria Prevention in Pregnancy. Int J Gynaecol Obstet (2019) 146(1):43–55. 10.1002/ijgo.12835 31050803

[22] Desai M Hill J Fernandes S Walker P Pell C Gutman J Prevention of Malaria in Pregnancy. Lancet Infect Dis (2018) 18(4):e119–32. 10.1016/S1473-3099(18)30064-1 29395997

[23] Salam RA Cousens S Welch V Gaffey M Middleton P Makrides M Mass Deworming for Soil-Transmitted Helminths and Schistosomiasis Among Pregnant Women: A Systematic Review and Individual Participant Data Meta-Analysis. Campbell Syst Rev (2019) 15(3):e1052. 10.1002/cl2.1052 PMC8356523 37131518

[24] Brocklehurst P Gordon A Heatley E Milan SJ. Antibiotics for Treating Bacterial Vaginosis in Pregnancy. Cochrane Database Syst Rev (2013) 1(1):CD000262. 10.1002/14651858.CD000262 PMC11307253 23440777

[25] Wingert A Pillay J Sebastianski M Gates M Featherstone R Shave K Asymptomatic Bacteriuria in Pregnancy: Systematic Reviews of Screening and Treatment Effectiveness and Patient Preferences. BMJ Open (2019) 9(3):e021347. 10.1136/bmjopen-2017-021347 PMC6429717 30872538

[26] Smaill FM Vazquez JC. Antibiotics for Asymptomatic Bacteriuria in Pregnancy. Cochrane Database Syst Rev (2019) 11(2):CD000490. 10.1002/14651858.CD000490.pub3 PMC6953361 31765489

[27] Iheozor-Ejiofor Z Middleton P Esposito M Glenny A-M. Treating Periodontal Disease for Preventing Adverse Birth Outcomes in Pregnant Women. Cochrane Database Syst Rev (2017) 6(6):CD005297. 10.1002/14651858.CD005297.pub3 28605006 PMC6481493

[28] Bi WG Emami E Luo Z-C Santamaria C Wei SQ. Effect of Periodontal Treatment in Pregnancy on Perinatal Outcomes: a Systematic Review and Meta-Analysis. J Matern Fetal Neonatal Med (2019) 0(0):3259–68. 10.1080/14767058.2019.1678142 31630597

[29] Ota E Hori H Mori R Tobe-Gai R Farrar D. Antenatal Dietary Education and Supplementation to Increase Energy and Protein Intake. Cochrane Database Syst Rev (2015) 11(6):CD000032. 10.1002/14651858.CD000032.pub3 PMC12634316 26031211

[30] Girard AW Olude O. Nutrition Education and Counselling provided during Pregnancy: Effects on Maternal, Neonatal and Child Health Outcomes. Paediatr Perinat Epidemiol (2012) 26(1):191–204. 10.1111/j.1365-3016.2012.01278.x 22742611

[31] Imdad A Bhutta ZA. Maternal Nutrition and Birth Outcomes: Effect of Balanced Protein-Energy Supplementation. Paediatr Perinat Epidemiol (2012) 26:178–90. 10.1111/j.1365-3016.2012.01308.x 22742610

[32] Peña-Rosas JP De-Regil LM Garcia-Casal MN Dowswell T. Daily Oral Iron Supplementation during Pregnancy. Cochrane Database Syst Rev (2015) 2015(7):CD004736. 10.1002/14651858.CD004736.pub5 PMC8918165 26198451

[33] Oh C Keats EC Bhutta ZA. Vitamin and Mineral Supplementation during Pregnancy on Maternal, Birth, Child Health and Development Outcomes in Low- and Middle-Income Countries: A Systematic Review and Meta-Analysis. Nutrients (2020) 12(2):491. 10.3390/nu12020491 PMC7071347 32075071

[34] Keats EC Haider BA Tam E Bhutta ZA. Multiple-micronutrient Supplementation for Women during Pregnancy. Cochrane Database Syst Rev (2019) 4(4):CD004905. 10.1002/14651858.CD004905.pub3 PMC6418471 30873598

[35] Das JK Hoodbhoy Z Salam RA Bhutta AZ Valenzuela-Rubio NG Prinzo ZW Lipid-based Nutrient Supplements for Maternal, Birth, and Infant Developmental Outcomes. Cochrane Database Syst Rev (2018) 8:CD012610. 10.1002/14651858.CD012610.pub2 30168868 PMC6513224

[36] Hofmeyr GJ Lawrie TA Atallah ÁN Torloni MR. Calcium Supplementation during Pregnancy for Preventing Hypertensive Disorders and Related Problems. Cochrane Database Syst Rev (2018) 1:CD001059. 10.1002/14651858.CD001059.pub3 PMC6517256 30277579

[37] Middleton P Gomersall JC Gould JF Shepherd E Olsen SF Makrides M. Omega-3 Fatty Acid Addition during Pregnancy. Cochrane Database Syst Rev (2018) 11:CD003402. 10.1002/14651858.CD003402.pub3 30480773 PMC6516961

[38] Duley L Meher S Hunter KE Seidler AL Askie LM. Antiplatelet Agents for Preventing Pre-eclampsia and its Complications. Cochrane Database Syst Rev (2019) 2019. 10.1002/14651858.CD004659.pub3 PMC6820858 31684684

[39] McMillan M Clarke M Parrella A Fell DB Amirthalingam G Marshall HS. Safety of Tetanus, Diphtheria, and Pertussis Vaccination during Pregnancy: A Systematic Review. Obstet Gynecol (2017) 129(3):560–73. 10.1097/AOG.0000000000001888 28178054

[40] Furuta M Sin J Ng ESW Wang K. Efficacy and Safety of Pertussis Vaccination for Pregnant Women – a Systematic Review of Randomised Controlled Trials and Observational Studies. BMC Pregnancy Childbirth (2017) 17(1):390. 10.1186/s12884-017-1559-2 29166874 PMC5700667

[41] Campbell H Gupta S Dolan GP Kapadia SJ Singh AK Andrews N Review of Vaccination in Pregnancy to Prevent Pertussis in Early Infancy. J Med Microbiol (2018) 67(10):1426–56. 10.1099/jmm.0.000829 30222536

[42] Macias Saint-Gerons D Solà Arnau I De Mucio B Arévalo-Rodríguez I Alemán A Castro JL Adverse Events Associated with the Use of Recommended Vaccines during Pregnancy: An Overview of Systematic Reviews. Vaccine (2020) 39:B12–B26. 10.1016/j.vaccine.2020.07.048 32972737

[43] Pryce J Richardson M Lengeler C. Insecticide-treated Bed Nets and Curtains for Preventing Malaria. Cochrane Database Syst Rev (2018) 11(11):CD000363. 10.1002/14651858.CD000363.pub2 30398672 PMC6418392

[44] Kahwati LC Clark R Berkman N Urrutia R Patel SV Zeng J Screening for Bacterial Vaginosis in Pregnant Adolescents and Women to Prevent Preterm Delivery: Updated Evidence Report and Systematic Review for the US Preventive Services Task Force. JAMA (2020) 323(13):1293–309. 10.1001/jama.2020.0233 32259235

[45] Peña-Rosas JP De-Regil LM Malave HG Flores-Urrutia MC Dowswell T. Intermittent Oral Iron Supplementation during Pregnancy. Ceylon Med J (2015) 46(4):132–5. 10.4038/cmj.v46i4.6440 12164031

[46] Christian P Jiang T Khatry SK LeClerq SC Shrestha SR West KP Jr. Antenatal Supplementation with Micronutrients and Biochemical Indicators of Status and Subclinical Infection in Rural Nepal. Am J Clin Nutr (2006) 83(4):788–94. 10.1093/ajcn/83.4.788 16600929

[47] Schulze KJ Mehra S Shaikh S Ali H Shamim AA Wu LS-F Antenatal Multiple Micronutrient Supplementation Compared to Iron-Folic Acid Affects Micronutrient Status but Does Not Eliminate Deficiencies in a Randomized Controlled Trial Among Pregnant Women of Rural Bangladesh. J Nutr (2019) 149(7):1260–70. 10.1093/jn/nxz046 31006806 PMC6602890

[48] Hofmeyr GJ Lawrie TA Atallah ÁN Duley L. Calcium Supplementation during Pregnancy for Preventing Hypertensive Disorders and Related Problems. Cochrane Database Syst Rev (2010) 1:CD001059. 10.1002/14651858.CD001059.pub3 20687064

[49] Buppasiri P Lumbiganon P Thinkhamrop J Ngamjarus C Laopaiboon M Medley N. Calcium Supplementation (Other Than for Preventing or Treating Hypertension) for Improving Pregnancy and Infant Outcomes. Cochrane Database Syst Rev (2015) 1:CD007079. 10.1002/14651858.CD007079.pub2 PMC10614032 25922862

[50] Bakouei F Delavar MA Mashayekh-Amiri S Esmailzadeh S Taheri Z. Efficacy of N-3 Fatty Acids Supplementation on the Prevention of Pregnancy Induced-Hypertension or Preeclampsia: A Systematic Review and Meta-Analysis. Taiwan J Obstet Gynecol (2020) 59(1):8–15. 10.1016/j.tjog.2019.11.002 32039806

[51] Hunter BM Harrison S Portela A Bick D. The Effects of Cash Transfers and Vouchers on the Use and Quality of Maternity Care Services: A Systematic Review. PLOS ONE (2017) 12(3):e0173068. 10.1371/journal.pone.0173068 28328940 PMC5362260

[52] Murray SF Hunter BM Bisht R Ensor T Bick D. Effects of Demand-Side Financing on Utilisation, Experiences and Outcomes of Maternity Care in Low- and Middle-Income Countries: a Systematic Review. BMC Pregnancy Childbirth (2014) 14(1):30. 10.1186/1471-2393-14-30 24438560 PMC3897964

[53] Parker SW Hernandez Prado B Ramirez Villalobos MD Moreno Macias H Laird NM Meneses Gonzalez F Resultados de la evaluacion externa del programa de desarrollo humano Oportunidades. Inter-American Development Bank (2004).

[54] Harris-Fry HA Paudel P Harrisson T Shrestha N Jha S Beard BJ Participatory Women’s Groups with Cash Transfers Can Increase Dietary Diversity and Micronutrient Adequacy during Pregnancy, whereas Women’s Groups with Food Transfers Can Increase Equity in Intrahousehold Energy Allocation. J Nutr (2018) 148(9):1472–83. 10.1093/jn/nxy109 30053188 PMC6118166

[55] Hoffman MK Goudar SS Kodkany BS Metgud M Somannavar M Okitawutshu J Low-dose Aspirin for the Prevention of Preterm Delivery in Nulliparous Women with a Singleton Pregnancy (ASPIRIN): a Randomised, Double-Blind, Placebo-Controlled Trial. Lancet (2020) 395(10220):285–93. 10.1016/S0140-6736(19)32973-3 31982074 PMC7168353

[56] Goldshtein I Nevo D Steinberg DM Rotem RS Gorfine M Chodick G Association between BNT162b2 Vaccination and Incidence of SARS-CoV-2 Infection in Pregnant Women. JAMA (2021) 326(8):728–35. 10.1001/jama.2021.11035 34251417 PMC8276131

[57] Abu Raya B Edwards KM Scheifele DW Halperin SA. Pertussis and Influenza Immunisation during Pregnancy: a Landscape Review. Lancet Infect Dis (2017) 17(7):e209–22. 10.1016/S1473-3099(17)30190-1 28433704

[58] Cuningham W Geard N Fielding JE Braat S Madhi SA Nunes MC Optimal Timing of Influenza Vaccine during Pregnancy: A Systematic Review and Meta-Analysis. Influenza Other Respir Viruses (2019) 13(5):438–52. 10.1111/irv.12649 31165580 PMC6692549

[59] Ellingson MK Dudley MZ Limaye RJ Salmon DA O’Leary ST Omer SB. Enhancing Uptake of Influenza Maternal Vaccine. Expert Rev Vaccin (2019) 18(2):191–204. 10.1080/14760584.2019.1562907 PMC6378696 30587042

[60] Krishnaswamy S Lambach P Giles ML. Key Considerations for Successful Implementation of Maternal Immunization Programs in Low and Middle Income Countries. Hum Vaccin Immunother (2019) 15(4):942–50. 10.1080/21645515.2018.1564433 30676250 PMC6605837

[61] Christian P Khatry SK West KP. Antenatal Anthelmintic Treatment, Birthweight, and Infant Survival in Rural Nepal. Lancet (2004) 364(9438):981–3. 10.1016/S0140-6736(04)17023-2 15364190

[62] Torrone EA Morrison CS Chen P-L Kwok C Francis SC Hayes RJ Prevalence of Sexually Transmitted Infections and Bacterial Vaginosis Among Women in Sub-saharan Africa: An Individual Participant Data Meta-Analysis of 18 HIV Prevention Studies. Plos Med (2018) 15(2):e1002511. 10.1371/journal.pmed.1002511 29485986 PMC5828349

[63] Bellad MB Hoffman MK Mallapur AA Charantimath US Katageri GM Ganachari MS Clindamycin to Reduce Preterm Birth in a Low Resource Setting: a Randomised Placebo-Controlled Clinical Trial. BJOG Int J Obstet Gynaecol (2018) 125(12):1601–9. 10.1111/1471-0528.15290 29790266

[64] Wang Z He Y Zheng Y. Probiotics for the Treatment of Bacterial Vaginosis: A Meta-Analysis. Int J Environ Res Public Health (2019) 16:E3859. 10.3390/ijerph16203859 31614736 PMC6848925

[65] Wang D Wang M Darling AM Perumal N Liu E Danaei G Gestational Weight Gain in Low-Income and Middle-Income Countries: a Modelling Analysis Using Nationally Representative Data. BMJ Glob Health (2020) 5(11):e003423. 10.1136/bmjgh-2020-003423 PMC7661366 33177038

[66] Members of an Expert Consultation on Nutritious Food Supplements for Pregnant and Lactating. Framework and Specifications for the Nutritional Composition of a Food Supplement for Pregnant and Lactating Women (PLW) in Undernourished and Low Income Settings. Seattle, Washington: Gates Open Research (2019).

[67] Christian P Nanayakkara-Bind A Schulze K Wu L LeClerq SC Khatry SK. Antenatal Micronutrient Supplementation and Third Trimester Cortisol and Erythropoietin Concentrations. Matern Child Nutr (2016) 12(1):64–73. 10.1111/mcn.12138 PMC6860105 25059838

[68] Christian P Khatry SK LeClerq SC Dali SM. Effects of Prenatal Micronutrient Supplementation on Complications of Labor and Delivery and Puerperal Morbidity in Rural Nepal. Int J Gynaecol Obstet (2009) 106(1):3–7. 10.1016/j.ijgo.2009.03.040 19368922

[69] Omotayo MO Dickin KL O’Brien KO Neufeld LM De Regil LM Stoltzfus RJ. Calcium Supplementation to Prevent Preeclampsia: Translating Guidelines into Practice in Low-Income Countries. Adv Nutr (2016) 7(2):275–8. 10.3945/an.115.010736 26980810 PMC4785477

[70] Li F Pei L Huang G Ye H. Influence of omega-3 Fatty Acid and Vitamin Co-supplementation on Metabolic Status in Gestational Diabetes: A Meta-Analysis of Randomized Controlled Studies. Eur J Obstet Gynecol Reprod Biol (2020) 247:191–7. 10.1016/j.ejogrb.2020.02.024 32145487

[71] Amirani E Asemi Z Asbaghi O Milajerdi A Reiner Ž Mansournia MA The Effects of omega-3 Fatty Acids Supplementation on Metabolic Status in Pregnant Women: a Systematic Review and Meta-Analysis of Randomized Controlled Trials. J Diabetes Metab Disord (2020) 19(2):1685–99. 10.1007/s40200-020-00558-5 33520859 PMC7843696

