# Supplementary material for: Pregnancy Interventions to Improve Birth Outcomes: What Are the Effects on Maternal Outcomes? A Scoping Review
Source: Int J Public Health. 2022 Nov 2;67:1604620. doi: 10.3389/ijph.2022.1604620 (PMC9666362; doi:10.3389/ijph.2022.1604620)
Supplement: Supplementary file 1 [file DataSheet1.docx]

**Supplementary Material: Pregnancy Interventions and Maternal Outcomes**

Table of Contents

[**Supplementary Table 1. Search strategy example: Hib vaccination in pregnancy (global, 2021).** 2](#_Toc116982146)

[**Supplementary Table 2. Full Search Results, by intervention and database (global, 2021).** 3](#_Toc116982147)

[**Supplementary Table 3. Characteristics of included review articles (global, 2021).** 5](#_Toc116982148)

# **Supplementary Table 1. Search strategy example: Hib vaccination in pregnancy (global, 2021).**

| **PubMed Database**  Search conducted 1/25/21 |
| --- |
| A. Population: Pregnant women |
| "pregnancy"[MeSH Terms] OR "pregnant women"[MeSH Terms] OR "mothers"[MeSH Terms] OR "maternal health"[MeSH Terms] (**933991**) |
| "pregnan*"[Text Word] OR "trimester*"[Text Word] OR "mother*"[Text Word] OR "matern*"[Text Word] OR "expecting wom*"[Text Word] OR "expecting mother*"[Text Word] OR "expectant wom*"[Text Word] OR "expectant mother*"[Text Word] OR "antenatal*"[Text Word] OR "prenatal*"[Text Word] OR "pre-natal*"[Text Word] OR "post conception*"[Text Word] OR "post conception*"[Text Word] OR "postconception*"[Text Word] (**1271621**) |
| "gestat*"[Text Word] AND ("women*"[Text Word] OR "woman*"[Text Word] OR "female*"[Text Word] OR "mother*"[Text Word] OR "matern*"[Text Word]) (**214955**) |
| 1 OR 2 OR 3 (**1307562**) |
| B. Publication type: Systematic Reviews, Meta-Analyses, Reviews of reviews |
| ("Meta-Analysis"[Publication Type] OR "Systematic Review"[Publication Type] OR "meta analys*"[Text Word] OR "meta analys*"[Text Word] OR "systematic review*"[Text Word] OR "review of review*"[Text Word]) NOT ("animals"[MeSH Terms] NOT "humans"[MeSH Terms]) (**322917**) |
| C. Intervention: Hib Vaccine |
| "haemophilus vaccines"[MeSH Terms] OR "haemophilus infections/prevention and control"[MeSH Terms] OR "haemophilus influenzae type b/immunology"[MeSH Terms] (**3779**) |
| “hemophilus vaccin*”[tw] OR “haemophilus influenzae vaccin*”[tw] OR “haemophilus vaccin*”[tw] OR “Haemophilus influenzae type b vaccin*”[tw] OR “Hib vaccin*”[tw] (**3592**) |
| 6 OR 7 (**4312**) |
| D. Combination of population, publication, and intervention |
| 4 AND 5 AND 8 (**1**) |
| **Embase Database**  Search conducted 01/25/2021 |
| A. Population: Pregnant women |
| 'pregnancy'/de OR 'pregnant woman'/de OR 'mother'/de OR 'maternal care'/de (**872705**) |
| pregnan*:ti,ab,kw OR trimester*:ti,ab,kw OR mother*:ti,ab,kw OR matern*:ti,ab,kw OR 'expecting wom*':ti,ab,kw OR 'expecting mother*':ti,ab,kw OR 'expectant wom*':ti,ab,kw OR 'expectant mother*':ti,ab,kw OR antenatal*:ti,ab,kw OR prenatal*:ti,ab,kw OR 'pre-natal*':ti,ab,kw OR 'pre natal*':ti,ab,kw OR 'post conception*':ti,ab,kw OR postconception:ti,ab,kw (**1186615**) |
| gestat* AND (women*:ti,ab,kw OR woman*:ti,ab,kw OR female*:ti,ab,kw OR mother*:ti,ab,kw OR matern*:ti,ab,kw) (**196378**) |
| 1 OR 2 OR 3 (**1460732**) |
| B. Publication type: Systematic Reviews, Meta-Analyses, Reviews of reviews |
| ('meta analysis'/de OR 'systematic review'/de OR 'meta analys*':ti,ab,kw OR 'systematic review*':ti,ab,kw OR 'review of review*':ti,ab,kw) NOT ('animal'/exp NOT 'human'/exp) (**473646**) |
| C. Intervention: Hib Vaccine |
| 'haemophilus influenzae type b vaccine'/exp OR 'hemophilus vaccin*':ti,ab,kw OR 'haemophilus influenzae vaccin*':ti,ab,kw OR 'haemophilus vaccin*':ti,ab,kw OR 'haemophilus influenzae type b vaccin*':ti,ab,kw OR 'hib vaccin*':ti,ab,kw (**5941**) |
| D. Combination of population, publication, and intervention |
| 4 AND 5 AND 6 (**9**) |
| **Cochrane Database of Systematic Reviews (CDSR)**  Search conducted on 01/25/2021 |
| A. Population: Pregnant women |
| [mh ^pregnancy] OR [mh ^"pregnant women"] OR [mh ^mothers] OR [mh ^"maternal health"] (**858**) |
| (pregnan* or trimester* or mother* or matern* or antenatal* or prenatal* or pre-natal* or "pre natal*" or post-conception* or "post conception*" or postconception*):ti,ab,kw (**1336**) |
| (expect* NEXT (mother* or woman* or women* or female*)):ti,ab,kw OR (gestat* and (women* or woman* or female* or mother* or matern*)):ti,ab,kw (**300**) |
| 1 OR 2 OR 3 (**1339**) |
| B. Publication type: Systematic Reviews, Meta-Analyses, Reviews of reviews |
| NOT ([mh animals] NOT [mh humans]) |
| C. Intervention: Hib Vaccine |
| [mh ^"haemophilus vaccines"] OR [mh ^"haemophilus infections"/PC] OR [mh ^"haemophilus influenzae type b"/IM] OR "hemophilus vaccin*":ti,ab,kw OR "haemophilus influenzae vaccin*":ti,ab,kw OR "haemophilus vaccin*":ti,ab,kw OR "haemophilus influenzae type b vaccin*":ti,ab,kw OR "hib vaccin*":ti,ab,kw (**7**) |
| D. Combination of population, publication, and intervention |
| 4 AND 5 AND 6 (**1**) |
| **Scopus Database**  Search conducted on 01/25/2021 |
| A. Population: Pregnant women |
| TITLE-ABS ( pregnan* OR trimester* OR mother* OR matern* OR antenatal* OR prenatal* OR pre-natal* OR "pre natal*" OR post-conception* OR "post conception*" OR postconception* ) (**1133277**) |
| TITLE-ABS ( gestat* AND ( women* OR woman* OR female* OR mother* OR matern* ) ) (**138365**) |
| 1 OR 2 (**1144960**) |
| B. Publication type: Systematic Reviews, Meta-Analyses, Reviews of reviews |
| DOCTYPE ( re ) AND SRCTYPE ( j ) AND NOT SUBJAREA ( ceng OR chem OR comp OR eart OR ener OR engi OR envi OR mate OR math OR phys OR vete ) (**2991214**) |
| C. Intervention: Hib Vaccine |
| TITLE-ABS ( "haemophilus influenzae type b vaccine*" OR "hemophilus vaccin*" OR "haemophilus influenzae vaccin*" OR "haemophilus vaccin*" OR "haemophilus influenzae type b vaccin*" OR "hib vaccin*" ) (**1716**) |
| D. Combination of population, publication, and intervention |
| 3 AND 4 AND 5 (**6**) |

# **Supplementary Table 2. Full Search Results, by intervention and database (global, 2021).**

| **Intervention** | **PubMed** | **Embase** | **Cochrane (CDSR)** | **Scopus** | **Total**^1^ |
| --- | --- | --- | --- | --- | --- |
| 1: Haemophilus influenzae type b (Hib) vaccination administered during pregnancy^2^ | 1 | 9 | 1 | 6 | 13 |
| 2: Influenza virus vaccination administered during pregnancy | 70 | 110 | 3 | 193 | 294 |
| 3: Tetanus Toxoid vaccination during pregnancy | 23 | 41 | 2 | 37 | 84 |
| 4: WASH interventions in pregnancy^3^ | 167 | 200 | 26 | 310 | 570 |
| 5: Provision of insecticide-treated bed nets in pregnancy | 18 | 21 | 0 | 50 | 71 |
| 6: Changing a two-dose IPTp regimen to more frequent IPTp dosing | 131 | 156 | 12 | 148 | 346 |
| 7: Changing the IPTp regimen from SP to DP |  |  |  |  |  |
| 8: Replacement of IPTp with ISTp (intermittent screening and treatment) |  |  |  |  |  |
| 9: Addition of an antibacterial antibiotic to the IPTp regimen^4^ |  |  |  |  |  |
| 10: Preventive anthelmintic treatment in pregnancy | 12 | 35 | 1 | 51 | 84 |
| 11: Clindamycin or metronidazole treatment of pregnant women with current BV | 137 | 218 | 18 | 48 | 348 |
| 12: Screening and treatment of STI other than HIV and syphilis^5^ | 70 | 8 | 2 | 51 | 126 |
| 13: Treatment of documented periodontal disease during pregnancy | 25 | 4 | 1 | 27 | 44 |
| 14: Treatment of documented deep caries or periapical periodontal disease during pregnancy^6^ | 14 | 31 | 0 | 1 | 41 |
| 15: Screening and treatment of asymptomatic bacteriuria in pregnancy | 42 | 35 | 7 | 109 | 150 |
| 16: Screening of tuberculosis in pregnancy in endemic areas^7^ | 18 | 5 | 2 | 21 | 44 |
| 17: Dietary education for undernourished pregnant women | 43 | 35 | 9 | 184 | 227 |
| 18: Iron-folic acid supplementation administered in pregnancy | 203 | 60 | 23 | 420 | 627 |
| 19: Replacement of iron-folic acid supplementation with MMN supplementation | 180 | 85 | 17 | 56 | 250 |
| 20: Provision of lipid-based nutrient supplements instead of multiple micronutrients | 5 | 6 | 0 | 4 | 8 |
| 21: Dietary supplementation with low and high dose calcium | 80 | 107 | 3 | 140 | 245 |
| 22: Provision of proteins and energy to undernourished pregnant women | 36 | 18 | 9 | 34 | 79 |
| 23: Supplementation with omega-3 fatty acids | 65 | 19 | 7 | 31 | 100 |
| 24: Conditional cash transfers (CCT) to pregnant women | 13 | 11 | 1 | 21 | 31 |
| 25: Unconditional cash transfers (UCT) to pregnant women^8^ | 2 | 2 | 0 | 0 | 3 |
| 26: Regular screening of maternal weight gain followed, if indicated, by dietary supplementation or other intervention^8^ | 21 | 27 | 6 | 72 | 101 |
| 27: Provision of low-dose aspirin during pregnancy | 76 | 13 | 2 | 21 | 97 |

^1^ Duplicates removed from total.

^2^ One Cochrane systematic review (Salam, 2015) assessed Hib vaccination during pregnancy, with only 1 included trail. The original trial (Glezen, 1992) and subsequent systematic review did not report any maternal outcomes and were therefore excluded from our review.

^3^ No relevant articles were identified for the effects of WASH interventions delivered at the household level during pregnancy on maternal outcomes.

^4^ No relevant articles were identified for the addition of an antibacterial antibiotic to the IPTp regimen on maternal outcomes.

^5^ No relevant articles were identified for the screening and treatment of STI other than HIV and syphilis on maternal outcomes.

^6^ No relevant articles were identified for the treatment of documented deep caries or periapical periodontal disease during pregnancy on maternal outcomes.

^7^ No relevant articles were identified for the screening of tuberculosis in endemic areas during pregnancy on maternal outcomes.

^8^ No relevant articles were identified for unconditional cash transfers to pregnant women on maternal outcomes.

^9^ No relevant articles were identified for the regular screening of maternal weight gain followed, if indicated, by dietary supplementation or other intervention in undernourished settings.

# **Supplementary Table 3. Characteristics of included review articles (global, 2021).**

| **Author, Year** | **Article type** | **Study designs** | **Settings** | **Intervention(s) description** | **Control/ comparator groups** | **Maternal outcomes** | **Quality assessment tool** | **Risk of bias** | **Quality of evidence**  **(GRADE)** |
| --- | --- | --- | --- | --- | --- | --- | --- | --- | --- |
| Bi, 2019 (34) | Systematic review and meta-analysis | 21 RCTs | UK (2), Colombia, USA (6), India (2), Chile (2), Australia, Hungary (2), Brazil (3), Iran | Interventions varied by study but included: scaling and root planning, scaling without root planning, oral hygiene instruction, prophylaxis, maintenance, and home supplies. Timing of treatment intervention varied by study. | No periodontal treatments. | - Pre-eclampsia, not defined. - Cesarean section - Gestational diabetes, not defined. | Cochrane risk assessment tool | 14 studies were evaluated as having high ROB due to lack of blinding. Risk of bias for other domains was overall low or unclear. | No GRADE assessments reported. |
| Brocklehurst, 2013 (29) | Cochrane review/ SRMA | 21 RCTs | Majority conducted in HICs. One study conducted in Indonesia. | Any antibiotic and dosage was included in the review. Regimens included oral metronidazole (9 trials); oral metronidazole + erythromycin (1); oral clindamycin (1); amoxicillin (1); vaginal metronidazole (1); intravaginal clindamycin (9). | Placebo or no treatment. | - Failure of test of cure, diagnosis varied by trial (Amsel or clinical criteria, Gram stain criteria, or abnormal Nugent score 4-10). - Postpartum infection, as defined by individual study authors. - Preterm pre-labor rupture of membranes (PPROM). - Side effects sufficient to stop or change treatment (not defined). - Side effects not sufficient to stop or change treatment (not defined). | Cochrane Handbook for Systematic Reviews of Interventions | Overall risk of bias was low, with 6 trials having a high ROB in 1 or two domains. The remaining 15 trials had low or unclear ROB for all domains. | No GRADE assessments reported. |
| Clark, 2020 (17) | Meta- analysis | 2 RCTs | Nepal and South Africa | Trivalent inactivated seasonal influenza vaccine administered in 2^nd^ or 3^rd^ trimester. | Saline placebo | - Maternal mortality by verbal autopsy report | No quality assessment |  |  |
| Das, 2018 (44) | Cochrane review/ SRMA | 4 RCTs | Ghana, Malawi, Burkina Faso, Bangladesh | In 3 trials, LNS was provided in 20 g/d sachets that contained 118 kcal and 22 micronutrients, including 20 mg iron. In one trial, the LNS supplement provided ~372 kcal/day and 16 micronutrients, including 35 mg of iron. Full composition included in article. | Control groups included IFA supplementation (60 mg iron + 400 mg folic acid) and MMS capsule. MMS composition not provided but micronutrient composition is described as like that of the LNS supplement. | - Weekly gestational weight gain (grams/week) from <20 weeks gestation until the time of delivery. - Anemia at term or near term (Hb<110 g/L). - Maternal death measured at 6 weeks postpartum. | Cochrane Handbook for Systematic Reviews of Interventions | Three studies were considered low ROB, one study was high ROB. Allocation concealment and blinding of assessors was not possible due to the nature of the intervention. | - Weekly gestational weight gain: MODERATE - Anemia: MODERATE - Maternal death: MODERATE |
| Desai, 2018 (27) | Meta-analysis within broader review | 4 RCTs for ISTp analysis | Kenya, Malawi, Ghana, the Gambia, Burkina Faso | Intermittent screening of malaria with rapid diagnostic tests and, if positive, treatment with artemisinin-based combination therapy (ACT). | Intermittent preventive treatment with sulfadoxine-pyrimethamine. | - Maternal parasitemia during pregnancy or at delivery. 2 trials used RDT, smear, PCR, or histology at delivery to determine malaria outcome. 1 trials used microscopy in late third trimester and another used placental histology at delivery. | Risk of bias and quality assessment not reported. |  |  |
| Duley, 2019 (54) | Cochrane Review/  SRMA | 77 RCTs. IPD was available for 36 trials. | Trials were conducted in 27 countries, the majority being high or upper middle-income, but some took place in LMICs. | Interventions varied by dose, duration, and use of other treatments. Most trials used aspirin alone, with doses ranging from 50 to 150 mg. Others used aspirin and dipyridamole, dipyridamole alone, heparin and dipyridamole, aspirin with micronutrients, ozagrel hydrochloride, and trapidil. | Placebo or no treatment | - Pre-eclampsia as defined by individual study authors. - Eclampsia, not defined. - Gestational hypertension defined as new hypertension with onset after 20 weeks’ gestation, using best available definition for every individual study. - Postpartum hemorrhage, defined as blood loss greater than 500 mL. - Placental abruption - Maternal death - Severe maternal morbidity, including eclampsia, liver failure, renal failure, disseminated intravascular coagulation, HELLP syndrome, stroke. | Cochrane Handbook for Systematic Reviews of Interventions | Most studies were either low ROB or unclear ROB. 25% of studies had high ROB for blinding. | - Preeclampsia: HIGH - Postpartum hemorrhage: MODERATE - Placental abruption: MODERATE - Other maternal outcomes did not have a reported GRADE score. |
| Gamble, 2007 (24) | Systematic Review | 5 RCTs | Thailand, Kenya (3 trials), Ghana | Insecticide treated nets, single-, double-, or family sized, provided at the household level. Insecticides used were permethrin (500 g/m^2^) or cyfluthrin. | No nets or untreated nets. | - Placental malaria defined as the presence of asexual parasitemia detectable by microscopy. - Hemoglobin (g/L). | Tool not described. | Composite score not provided. | Trial quality was assessed as adequate, inadequate, or unclear based on the methods used to generate the allocation sequence and allocation concealment, but no composite grades were provided. |
| Girard & Olude, 2012 (36) | Systematic Review and Meta-Analysis | 34 studies;  16 RCTs and  18 quasi-experimental/ non-randomized studies. | 11 studies conducted in LMICs, 23 in HICs. | Authors identified three intervention types: nutrition education as sole intervention (15 studies); package of health education messages, including nutrition (6 studies); nutrition education with additional food or micronutrient supplements (13 studies). | Comparison groups did not receive nutrition counselling or the system of delivery, number of sessions and/or intensity differed. | - Gestational weight gain (kg) - Anemia in 3^rd^ trimester or at delivery. Cut-off not defined. | Child Health Epidemiology Reference Group adaptation of the Grading of Recommendations, Assessment, Development and Evaluation (GRADE) technique | Ten studies were given very low scores, meaning a high risk of bias; nine were given low scores, fourteen were given moderate scores, and only one had a high score, meaning low risk of bias. | - Gestational weight gain: LOW - Anemia: LOW |
| Hofmeyr, 2018 (46) | Cochrane Review/  SRMA | High-dose calcium studies were all RCTs. Low dose calcium studies included RCTs and quasi-randomized designs. | Philippines, Iran, Trinidad, Argentina, China, Australia, Colombia, Gambia, India, Ecuador, Hong Kong, Indonesia, USA | 3 intervention types:  1. Daily high-dose (>1g) calcium started before 34 weeks.  2. Daily low-dose (<1g) calcium.  3. Comparison of different dosages of calcium. | Compared high-dose to placebo only.  Compared low-dose to placebo, no treatment, or high-dose calcium. | - High blood pressure as defined by trial authors, with or without proteinuria. - Pre-eclampsia: high blood pressure with significant proteinuria, as defined by trial authors. - Eclampsia (not defined) - Maternal death or serious morbidity included death; eclampsia; renal failure; syndrome of hemolysis, elevated liver enzymes and low platelets (HELLP syndrome); and admission to intensive care. - Placental abruption - Cesarean section | Assessed ROB using Cochrane Handbook for Systematic Reviews of Interventions | 23 studies had moderate risk of bias in at least one domain. 11 studies had high risk of bias for at least one domain; only four studies had low risk of bias across all six domains. | - Pre-eclampsia: LOW - HELLP syndrome: HIGH - GRADE scores for other outcomes were not provided. |
| Iheozor-Ejiofor, 2017 (33) | Cochrane review/ SRMA | 15 RCTs | North America (5 trials); South America (4); Europe (3); Asia (2); Australia (1) | Periodontal treatment, including scaling, root planning, polishing, and/or surgery. Sometimes combined with counselling on oral hygiene, antiseptic oral agents, topical or systemic antimicrobial therapies. | No treatment or alternative treatment (less intensive). | - Periodontal outcomes included probing depth, plaque index, bleeding on probe, and clinical attachment level as defined by individual study authors | Cochrane Handbook for Systematic Reviews of Interventions | All trials were considered to have high ROB, due to lack of blinding and imbalance in baseline characteristics of studies. | - GRADE scores not determined for periodontal outcomes. |
| Imdad & Bhutta, 2012 (37) | Systematic review and meta-analysis | 16 total studies. 13 RCTs and 2 quasi-randomized studies. One before and after design. | 8 studies conducted in LMICs, 8 studies conducted in HICs. | Balanced protein energy supplementation defined as nutritional supplementation during pregnancy in which protein provided less than 25% of total energy content. Supplements varied in format (energy drinks, biscuits, pastes, powdered milk, etc.) and some were fortified with additional micronutrients. | Comparison groups varied: routine diet (no intervention); placebo pills, micronutrient tablets; placebo drinks. | - Weekly gestational weight gain (g/week). - Pre-eclampsia (not defined). | Assessed quality using GRADE criteria but did not provide summary information on overall risk of bias. | No risk of bias summary information found. | Did not provide GRADE assessments for maternal outcomes. |
| Kayentao, 2013 (25) | Systematic review and meta-analysis | 7 RCTs | Kenya, Malawi, Zambia, Burkina Faso, Mali, Tanzania | 3 doses of or monthly intermittent preventive treatment with sulfadoxine-pyrimethamine (IPTp-SP) throughout pregnancy. Intake was supervised in all trials. | 2 doses of IPTp-SP. | - Hemoglobin (g/dL) measured at term or delivery. - Anemia (<11 g/dL) measured at term or delivery. - Moderate/severe anemia (<8-6 g/dL) measured at term or delivery. - Maternal parasitemia identified in peripheral blood at delivery. - Placental malaria (all species) identified by microscopy. | Cochrane risk of bias tool. | Two trials were considered to be of low quality due to high ROB in 3 or more domains. Only one trial had low or unclear ROB across all domains. | GRADE scores by outcome were not reported. |
| Keats, 2019 (41) | Cochrane Review/  SRMA | 20 RCTs | 19 trials conducted in LMICs | Daily multiple-micronutrient supplements containing 4 or more micronutrients, including iron and folic acid. Most trials started supplementation at enrollment and duration went through childbirth or later. Timing of enrollment varied by trial. | Daily iron supplements with or without folic acid. | - Anemia (Hb <110g/L) in the 3^rd^ trimester - Maternal death | Assessed ROB using Cochrane Handbook for Systematic Reviews of Interventions | Overall risk of bias was low across the six domains. 9 studies had low or unclear ROB across all domains.  7 studies had one or two domains with high ROB. | Did not provide GRADE score for maternal outcomes |
| Middleton, 2018 (48) | Cochrane Review/  SRMA | 70 RCTs | Trials conducted in mostly high and upper-middle income countries. 16 in USA, 8 in Iran, 6 in the UK, 4 in the Netherlands and 4 in Denmark. Others in Australia, Spain, Chile, Egypt, Germany, Italy, Brazil, Sweden, Angola, Bangladesh, Canada, Croatia, Finland, Mexico, Norway, South Africa, Taiwan, Turkey, and Venezuela. | Omega‐3 long-chain poly-unsaturated fatty acids (LCPUFA) in the form of supplements or food. Delivered as stand alone, or with a cointervention (food or nutritional counseling). | Placebo or no omega-3 fatty acids. | - High blood pressure without proteinuria, as defined by individual study authors. - Pre-eclampsia, defined as hypertension with proteinuria. - Eclampsia (not defined). - Gestational diabetes (not defined). - Anemia (not defined). - Gestational weight gain (kg) - Preterm pre-labor rupture of membranes (PPROM) - Pre-labor rupture of membranes (PROM) - Any adverse event, as defined by individual study authors. - Serious adverse events, not defined. - Maternal death - Cesarean section - Postpartum hemorrhage - Postpartum depression (EPDS) | Assessed ROB using Cochrane Handbook for Systematic Reviews of Interventions | Three studies had low risk of bias across all domains. More than half (33 studies) had high risk of bias in at least one domain. There was no assessment of bias for 3 studies. Overall, risk of bias was low to unclear across most domains. There was considerable ROB for incomplete outcome data (attrition bias). | Pre-eclampsia: LOW  Serious adverse events: LOW  Postnatal Depression: LOW  GRADE scores for other maternal outcomes not reported. |
| Oh, 2020 (39) | Systematic review and meta-analysis | 34 studies. RCTs and quasi-experimental designs included. | East Asia and the Pacific (12 studies); Middle-east and North Africa (13 studies); sub-Saharan Africa (18 studies); South Asia (19 studies); Latin America and the Caribbean (7 studies). | Assessed various micronutrient supplements administered in pregnancy. We extracted data from the iron, IFA, and multiple micronutrient sections. Iron supplementation included daily iron supplements. IFA included daily iron and folic acid supplementation. MMS included daily supplements with 3 or more micronutrients. A third of the studies used UNIMMAP formulation.  Start and duration of supplementation varied across trials. | Iron supplementation was compared to placebo or no treatment; IFA was compared to folic acid alone; MMS was compared to IFA. | - Serum/plasma retinol concentration (umol/L) - Serum/plasma zinc concentration (nmol/L) - Serum/plasma vitamin B12 concentration (pmol/L) - Hemoglobin concentration (g/L) - Serum/plasma ferritin concentration (ug/L) - Pre-eclampsia or eclampsia | Assessed using Cochrane Risk of Bias (ROB) tool for RCTs and the Cochrane Effective Practice and Organization of Care (EPOC) guidelines for controlled before/after studies and interrupted time series studies. | Risk of bias for included studies was generally low, with 75% considered low ROB for four domains and at least 50% of judgements as low ROB for three domains of quality assessment. | Did not provide GRADE for these maternal outcomes. |
| Olaleye, 2019 (26) | Systematic review and meta-analysis | 3 RCTs | Uganda, Kenya, Malawi | Assessed three intervention designs:  1. 3 doses or  2. monthly IPTp with dihydroartemisinin-pyrimethamine (DP). Only the initial doses of each drug were given as directly observed therapy.  3. Intermittent screening and treatment in pregnancy (ISTp) with DP. Rapid diagnostic tests were used to screen for malaria, timing not specified. | IPTp with sulfadoxine-pyrimethamine | - Clinical malaria during pregnancy, defined as presence of asexual parasites and fever during pregnancy. - Placental malaria, defined by individual study authors. - Maternal peripheral malaria at delivery, as defined by individual study authors. - Anemia (Hb <11 g/dL) - Maternal serious adverse events (SAEs) as defined by individual study authors. | Cochrane risk of bias tool | 3 trials considered to have an overall low risk of bias. | For IPTp-DP:   - Clinical malaria: MODERATE - Placental malaria: MODERATE - Peripheral malaria: MODERATE - Anemia: MODERATE - SAEs: LOW   For ISTp-DP:   - Clinical malaria: LOW - Placental malaria: HIGH - Peripheral malaria: HIGH - Anemia: MODERATE - SAEs: LOW |
| Ota, 2015 (35) | Cochrane Review/  SRMA | 17 RCTs total:  5 RCTs for nutritional education  12 RCTs for balanced energy/ protein supplementation | 7 trials conducted in HICs. 10 trials conducted in LMICs: the Gambia, India, Burkina Faso, Indonesia, Colombia, South Africa, and Bangladesh | Nutrition education: Specific dietary education to pregnant women to increase protein and energy intake. Most trials provided a series of in-home or group counselling sessions by trained staff.  BEP: Interventions included food or drink supplements providing additional energy and protein, some with added micronutrients. One trial provided vouchers for milk. Nutritional composition varied by trial. Most interventions were initiated after the 1^st^ trimester. | Nutrition education: No intervention or consultation without nutrition counselling.  BEP: Various control/ comparison groups. Some provided no intervention, some provided micronutrient tablets, some provided supplements with smaller macronutrient content. | - Protein intake (g/day): dietary assessment methods not described. - Energy intake (kcal/day) - Gestational weight gain (kg) - Pre-eclampsia (defined by trialists) | Cochrane Handbook for Systematic Reviews of Interventions | 4 trials had a low or unclear risk of bias for all categories. 13 trials had high ROB for at least one type of bias, mostly due to the inability to blind participants to intervention. | - Gestational weight gain: VERY LOW - GRADE scores not provided for other maternal outcomes. |
| Peña-Rosas, 2015 (38) | Cochrane review/ SRMA | 61 trials were included but 44 studies contributed data. These included RCTs and quasi-randomized designs. | Europe (24); Americas (11); sub-Saharan Africa (4); Iran (4); East Asia (6); Australia (3); South and SE Asia (8) | In most trials. supplementation started before 20 weeks’ gestation, but for 13 trials, supplementation started at or after 20 weeks. Daily dosage ranged from 9 mg to 900 mg elemental iron. Majority provided 60-100 mg. For those with additional folic acid, doses ranged from 10 µg to 5000 µg. | Supplements without iron, placebo, or no treatment. | - Anemia at term, Hb <110 g/L at 37 weeks’ gestation or more. - Iron deficiency at term, as defined by trialists, based n any indicator of iron status at 37 weeks’ gestation or more. - Severe anemia, Hb <70 g/L at any time during 2^nd^ or 3^rd^ trimester. - Any side effects reported throughout intervention period. | Cochrane Handbook for Systematic Reviews of Interventions | A majority of studies had a high or unclear risk of bias in at least one domain. Over 25% of studies had a high ROB for blinding and incomplete outcome data. | - Anemia: LOW - Iron deficiency: LOW - Severe anemia: VERY LOW - Side effects: LOW |
| Quach, 2020 (16) | Systematic review and meta-analysis | 19 total studies.  5 RCTs; 12 cohort studies; 2 case control studies | 7 studies conducted in Asia; 6 in US; 2 in Europe; 2 in Oceania; and 2 in Africa | Trivalent inactivated influenza vaccine; timing not specified.  Also assessed monovalent H1N1 vaccine but not included in this report. | Saline placebo; quadrivalent meningococcal conjugate vaccine; 23-valent pneumococcal polysaccharide vaccine. | - Laboratory-confirmed influenza by PCR - Influenza-like illness (not defined) - Respiratory illness (not defined) | For clinical trials: Cochrane risk of bias instrument  For observational studies: Newcastle-Ottawa quality assessment scale | Clinical trials  4 low ROB  1 high ROB  Observational studies  6 studies low ROB  2 medium ROB  4 high ROB | Did not perform GRADE assessment |
| Salam, 2019 (28) | Systematic review and meta-analysis | 7 RCTs identified but only 3 trials provided individual-participant data for the analyses. | Philippines, Tanzania, Uganda, | Treatment and dose varied: Albendazole (Single dose 400 mg at term, 2 doses of 200 mg in second trimester)  Praziquantel (40 mg/kg at one dose, or 60 mg/kg at 2 doses)  Mebendazole (500 mg)  Ivermectin | Placebo or nutritional tablet | - Anemia at term (Hb < 11 g/dL) - Infection intensity- T. trichiura (any infection) - Infection intensity- Hookworm (any infection) | Assessed ROB using Cochrane risk of bias tool | One trial had an overall low ROB. Two other trials had high ROB in one domain: allocation concealment and incomplete outcome data, respectively. | - Anemia: MODERATE - T.trichiura intensity : MODERATE - Hookworm intensity: MODERATE |
| Smaill & Vazquez, 2019 (31) | Cochrane review/ SRMA | 15 studies, including randomized and quasi-randomized trials. | HICs only. | Any antibiotic regimen. Antibiotics were given in a single dose for 3-7 days, for 3 weeks, for 6 weeks, until delivery, or up to 6 weeks after delivery. Antibiotics included: sulphonamides, tetracycline, methenamine, nitrofurantoin, and ampicillin. | Placebo or no treatment. | - Pyelonephritis (kidney infection), not defined. - Persistent bacteriuria, defined as a positive culture at delivery, at the last prenatal visit, or 6 weeks to 3 months postpartum. | Cochrane Handbook for Systematic Reviews of Interventions | Most studies had a high risk of bias in at least two domains. Many studies also had an unclear risk of bias for most of the domains. Only one study had low ROB across all domains. | - Pyelonephritis: LOW - No GRADE score for persistent bacteriuria |
| Vygen-Bonnet, 2020 (22) | Systematic review | 14 studies included for vaccine safety: 3 RCTs and 11 non-randomized studies. | Belgium, UK, Canada, New Zealand, Vietnam, USA | Tetanus-diphtheria-acellular pertussis (Tdap); Tdap-IPV. Timing of vaccination not specified. | Saline placebo; no treatment; tetanus toxoid vaccine | - Fever >38° after vaccination. Assessed with medically attended fever in 3 days post-vaccination. - Hypertension: ICD10 and not defined - Pre-eclampsia: Varying definitions from individual studies. - Eclampsia: ICD10, ICD9, others. - Chorioamnionitis: Assessed with ICD9, ICD10, others/not defined. | For RCTs: Cochrane risk of bias tool  For non-randomized studies: ROBINS-I tool | RCTs  1 high ROB  1 moderate ROB  1 low ROB  Observational  8 serious ROB  3 critical ROB | - Fever: LOW - Pre-eclampsia: LOW - Chorioamnionitis: LOW - Hypertension and Eclampsia did not receive a GRADE score. |
| Wingert, 2019 (32) | Systematic review and meta-analysis | 25 studies: 11 RCTs, 4 controlled clinical trials, 5 cohort studies, 5 cross-sectional.  For the comparison of screening vs. no screening, data came from 4 cohort studies. | HICs: France, Spain, Turkey, USA | For screening effectiveness, interventions included screening at first prenatal visit only (3 studies), or at every prenatal visit (1 study). Mid-stream urine cultures were used to test for ASB, and the criteria used for a positive test was >10^8^CFU/L or not reported. | No screening for ASB. | - Pyelonephritis, or kidney infection, occurring up until delivery. Definitions or diagnosis not described. | For observational studies on screening effectiveness, the Newcastle-Ottawa Quality Assessment Scale was used. | 3 studies considered unclear ROB and 1 study considered low ROB. | - Pyelonephritis: VERY LOW |
